# Benzothiazole-Derived Compounds as Potential Drugs for the Treatment of Type 2 Diabetes: A Descriptive Review

**DOI:** 10.3390/ijms27198863

**Published:** 2026-10-05

**Authors:** Ana María Correa-Basurto, Miguel Valdes, Laura Cristina Cabrera-Pérez, Natalia Reyes Vallejo, Alejandro Cruz, Martha Cecilia Rosales-Hernández, Juan Andres Alvarado-Salazar, Jessica Elena Mendieta-Wejebe

**Affiliations:** 1 Laboratorio de Biofísica y Biocatálisis, Sección de Estudios de Posgrado e Investigación, Escuela Superior de Medicina, Instituto Politécnico Nacional, Plan de San Luis y Salvador Díaz Mirón s/n, Casco de Santo Tomás, Miguel Hidalgo, Mexico City 11340, Mexico; anacb2025@gmail.com (A.M.C.-B.); valdesguevaramiguel@gmail.com (M.V.); reyes.natalia4@gmail.com (N.R.V.); marcrh2002@yahoo.com.mx (M.C.R.-H.); 2 Unidad de Investigación Médica en Farmacología, UMAE Hospital de Especialidades 2 Piso CORSE Centro Médico Nacional Siglo XXI, Instituto Mexicano del Seguro Social, Av. Cuauhtémoc 330, Col. Doctores, Ciudad de México 06725, Mexico; 3 Laboratorio de Inmunología, Departamento de Sistemas Biológicos, Universidad Autónoma Metropolitana Unidad Xochimilco, Calz. del Hueso 1100, Coapa, Villa Quietud, Coyoacán, Ciudad de México 04960, Mexico; 4 Laboratorio de Química Supramolecular y Nanociencias, Unidad Profesional Interdisciplinaria de Biotecnología, Instituto Politécnico Nacional, Av. Acueducto s/n, Barrio La Laguna Ticomán, Ciudad de México 07340, Mexico; uva6210@yahoo.com.mx (L.C.C.-P.); alcralmx@hotmail.com (A.C.); 5 Licenciatura de Química Farmacéutico Biológica, Área Farmacéutica, Facultad de Estudios Superiores (FES)-Zaragoza, Universidad Nacional Autónoma de México (UNAM), Batalla 5 de mayo s/n, Ejército de Oriente, Iztapalapa, Ciudad de México 09230, Mexico; 6 Academia de Farmacología y Toxicología, Licenciatura de Química Farmacéutico Biológica, Facultad de Estudios Superiores (FES)-Zaragoza, Universidad Nacional Autónoma de México (UNAM), Batalla 5 de mayo s/n, Ejército de Oriente, Iztapalapa, Ciudad de México 09230, Mexico

**Keywords:** benzothiazole, type 2 diabetes, antidiabetic agents, medicinal chemistry, drug development

## Abstract

Type 2 diabetes (T2D) is a multifactorial metabolic disease in which hyperglycemia coexists with insulin resistance, *β*-cell dysfunction, dyslipidemia, and progressive microvascular and macrovascular complications. The structural versatility of the benzothiazole scaffold has enabled the development of a diverse array of preclinical compounds capable of modulating multiple diabetes-relevant targets. This review critically analyzes the available evidence on benzothiazole-derived compounds exhibiting antidiabetic or other diabetes-related pharmacological activities. No lower publication-date limit was imposed, and the literature included in the review ranged from 1997 to March 2026, encompassing in silico, in vitro, ex vivo, and in vivo evidence. The compounds are discussed according to their primary molecular targets, including α-glucosidase and α-amylase, dipeptidyl peptidase-4 (DPP-4), peroxisome proliferator-activated receptor -γ (PPAR-γ), protein tyrosine phosphatase 1B (PTP1B), glycogen phosphorylase, 11β-hydroxysteroid dehydrogenase 1 (11β-HSD1), aldose reductase, AMPK, and other emerging metabolic targets. Structure–activity relationships (SAR) are highlighted, demonstrating that C-2 functionalization, substitution at the C-5 and C-6 positions, halogenation, the presence of electron-donating or electron-withdrawing groups, and hybridization with thiazolidinedione, rhodanine, triazole, oxadiazole, and other pharmacophores can markedly alter potency, selectivity, and metabolic effects. Various derivatives have matched or outperformed reference agents such as acarbose, glibenclamide, pioglitazone, or aldose reductase inhibitors in preclinical assays. However, a direct comparison between studies is limited by the heterogeneity of models, endpoints, units, and dosing regimens, and clinical efficacy for T2D has not yet been established for this chemical class. Overall, the benzothiazole scaffold emerges not as a universal antidiabetic pharmacophore, but as a highly adaptable platform for the rational design of multitarget compounds.

## 1. Introduction

Diabetes is one of the major diseases of global concern, since inadequate care can lead to various complications, including disabling conditions or premature death, generating an enormous economic burden on healthcare systems, affected individuals, and their families.

Diabetes comprises a heterogeneous group of metabolic disorders characterized mainly by a state of hyperglycemia (blood glucose levels above 126 mg/dL) associated with other alterations in carbohydrate, protein, and/or lipid metabolism, caused by defects in insulin secretion and/or decreased action of this hormone [1,2]. These alterations are reflected by the exacerbation of biological and pathological processes during the disease that influence the function of the pancreatic β-cells, causing their autoimmune destruction, and that lead to a high risk of developing complications, these being hyperglycemia, glucotoxicity, lipotoxicity, autoimmunity, inflammation, an imbalance in adipokine concentrations, and decreased incretin effects, as well as insulin resistance and oxidative stress [3,4].

According to the American Diabetes Association (ADA), diabetes can be broadly classified into the following categories [5,6]: (a) type 1 diabetes (T1D), of autoimmune origin, due to the destruction of pancreatic β-cells and usually leading to absolute insulin deficiency; (b) type 2 diabetes (T2D), caused by a progressive loss of adequate insulin secretion from pancreatic β-cells, often in the context of insulin resistance; (c) gestational diabetes, which can be diagnosed in the second or third trimester of pregnancy, which is characterized by hyperglycemia values and which, despite being higher than normal, are lower than those established to diagnose diabetes; (d) specific types of diabetes, linked to other causes, e.g., monogenic diabetes syndromes, diseases of the exocrine pancreas, and diseases induced by drugs or chemicals. Despite several factors that cause this disease, it is alarming that T2D, associated with poor eating habits and a sedentary lifestyle, is the most prevalent type of diabetes, accounting for 90% of all cases worldwide [1,7].

Due to the significant increase in diabetes, there are currently numerous classes of antihyperglycemic drugs approved to treat hyperglycemia in patients with T2D. However, it is well known that many of these have adverse effects and reported pharmacological resistance to some antidiabetic treatments, as well as the difficulty in managing this disease when it is associated with other metabolic disorders. Therefore, the development of new alternatives based on structural prototypes is one of the main areas of interest for science making it necessary to continue research and apply new technological innovations to identify effective drugs [8,9].

Among the latest trends in pharmaceutical chemistry research is the growing popularity of molecular hybridization for new drug development, which involves the combination of two or more pharmacophoric fractions of different biologically active substances to create a new hybrid molecule that is potent and has higher efficacy and affinity compared to the standard drug. Considering the above, it has been reported that various compounds incorporating fused heterocyclic and heterocyclic rings play an important role in the design and synthesis of antidiabetic agents, mainly those containing nitrogen, sulfur, and oxygen atoms, due to their diverse and promising pharmaceutical applications [10]. In this regard, benzothiazoles, which belong to the class of bicyclic heteroaromatics, have attracted considerable interest in medicinal chemistry as molecules of great biological and therapeutic utility, because they have a valuable and broad spectrum of biological activities, such as anticancer, antimicrobial, antiviral, anticonvulsant, antiobesity, anti-inflammatory, and antidiabetic, among others [2,11].

Previous reviews have highlighted the importance of heterocyclic compounds in the development of potential antidiabetic agents [10], while benzothiazole-based molecules have been the subject of extensive reviews from a broader medicinal chemistry perspective, covering a wide range of biological and therapeutic activities [11]. Although these studies demonstrate the pharmacological versatility of the benzothiazole scaffold, their scope does not specifically focus on integrating the various benzothiazole derivatives based on the molecular targets and metabolic pathways involved in T2D. Concurrently, individual experimental studies have shown that suitably substituted benzothiazole derivatives can interact with specific diabetes-related targets, such as α-glucosidase, underscoring the relevance of this scaffold in antidiabetic drug discovery [12].

Consequently, this review aims to provide an up-to-date, target-oriented analysis of benzothiazole derivatives with potential relevance to T2D and the pharmacological activity that they exhibit. Unlike more general reviews on heterocyclic or benzothiazole-containing compounds, this work integrates evidence from in silico, in vitro, ex vivo, and in vivo studies, organizing the available information according to key molecular targets involved in glucose homeostasis, insulin signaling, metabolic regulation, and diabetes-associated complications. Special attention is paid to structure–activity relationships, substitution patterns, molecular hybridization strategies, and the experimental evidence supporting the pharmacological effects of these compounds. Thus, the review seeks to elucidate the contribution of the benzothiazole scaffold to antidiabetic activity while identifying limitations and translational gaps in the currently available evidence.

## 2. Literature Search Strategy and Scope

An extensive and iterative literature search was conducted using multiple bibliographic databases and academic search platforms including PubMed, Scopus, Web of Science, SciELO, Redalyc, the Directory of Open Access Journals (DOAJ), and Google Scholar to identify publications concerning benzothiazole-derived compounds that exhibited antidiabetic activity or pharmacological relevance to T2D. Given that the aim of this descriptive review was to provide a comprehensive overview of the development and pharmacological potential of benzothiazole-based compounds in this field, no predefined lower limit was set for the publication date. Instead, the search was deliberately performed without a start-year restriction to identify early relevant reports and track the subsequent evolution of this chemical scaffold. The literature search was updated to March 2026. Consequently, the selected literature ranged from 1997, the year of the oldest relevant publication retrieved, to March 2026, representing the most recent evidence included at the time of the final update.

Searches were carried out iteratively using combinations of terms related to the benzothiazole scaffold, T2D, antidiabetic activity, and the molecular targets addressed in this review. Representative search terms included “benzothiazole,” “benzothiazole derivatives,” “type 2 diabetes,” “antidiabetic,” “hypoglycemic,” “insulin resistance,” “α-amylase,” “α-glucosidase,” “DPP-IV,” “PPAR,” “PTP-1B,” “glycogen phosphorylase,” “11*β*-HSD1,” “aldose reductase,” “AMPK,” and “obesity.” These terms were used individually or in various combinations, depending on the specific target, pharmacological mechanism, or biological activity under analysis. Additionally, further relevant studies were identified through a manual review of the reference lists of the selected articles. The review focused primarily on original research articles describing the design, synthesis, characterization, biological evaluation, structure–activity relationships, molecular modeling, or pharmacological effects of benzothiazole-containing compounds relevant to T2D. Evidence from in silico, in vitro, ex vivo, and in vivo studies was considered.

For clarity, the evidence analyzed throughout this review is classified according to the experimental level at which it was obtained: computational modeling, molecular docking, molecular dynamics, and related computational approaches are described as in silico evidence; enzyme or cell-based experimental assays as in vitro evidence; studies conducted on isolated tissues or biological samples outside the intact organism as ex vivo evidence; and animal studies as in vivo evidence. No clinical studies evaluating benzothiazole-derived compounds specifically for diabetes treatment were identified.

Additionally, review articles, clinical guidelines, and reference sources were consulted to support the sections addressing the pathophysiology of T2D, currently available pharmacological treatments, molecular targets, and the medicinal chemistry of benzothiazoles.

As this work was conceived as a descriptive rather than a systematic review, the evidence was synthesized narratively and organized according to the primary molecular or biological target investigated. Direct quantitative comparisons between studies were not always possible due to differences in experimental models, assay conditions, endpoints, doses, concentrations, and reported units. Consequently, results were interpreted within their respective experimental contexts, emphasizing recurring structure–activity relationships and the contribution of the benzothiazole scaffold to the design of potential antidiabetic agents.

## 3. Diabetes

In 2024, diabetes affected 589 million (11.11%) of the global adult population, with an estimated 635 million people having impaired glucose tolerance and 488 million having impaired fasting glucose. In addition, the disease is projected to affect 853 million (12.96%) people aged 20–79 years by 2050, equivalent to an increase of 45%. Prevalence peaked at 24.79% among those aged 75–79 years and was 23.72% in adults aged >65 years; moreover, it was higher in males than females (11.55% vs. 10.68%, respectively). Also, this disease was more prevalent in urban than rural areas (12.26% vs. 9.23%, respectively), and middle-income countries had the highest prevalence (11.46%), followed by high-income (10.21%) and low-income (7.47%) countries [13,14,15,16].

As a definition, diabetes is a complex metabolic disease characterized by an increase in the blood glucose concentration, resulting in metabolic disorders, and micro and macrovascular damage that gradually interrupt the structure and functioning of multiple organs, such as heart, eyes, kidneys, and nerves [17,18].

Type 1 diabetes (T1D) is caused by an exacerbated inflammatory process due to the infiltration of cells that can trigger an autoimmune process that atrophies pancreatic cells, causing no insulin production, which is also known as autoimmune insulitis [19]. On the other hand, type 2 diabetes (T2D) is defined as a metabolic disorder of glucose homeostasis caused by the secretion of insulin in amounts lower than those required; it is characterized by chronically elevated glycemic levels and alterations in lipid metabolism [20]. It also leads to resistance to the action of insulin on peripheral tissues and to deterioration in the suppression of glucagon secretion in response to glucose intake [21,22]. Recent advances describe this pathology as a metabolic disorder of multiple etiologies, and it has been found that multiple organs are involved in the pathogenesis of T2D. The proposed pathophysiological mechanisms are diverse, either because they are derived from the analysis from different points of view of a common pathogenic mechanism or because there are specific mechanisms of the type of tissue. In recent years, several studies have been reported in the pancreas, skeletal muscle, heart, adipose tissue, and liver, as they are related to obesity, insulin resistance, and T2D. This has allowed us to gain important information for the understanding of the mechanisms that lead to the development of this disease and its micro and macrovascular complications. With these advances, it will be possible to obtain better therapeutic approaches and biomarkers for diagnosis, monitoring, and the evaluation of potential drugs for T2D treatment [23], since knowledge of the molecular bases of diabetes denotes a better interpretation of this entity and its relationship with other pathologies, because, cardiovascular diseases are not the only ones related to the development of diabetes; it has also been found that they increase the risk of developing dementia and other neurodegenerative diseases by 1.9% [14,24,25,26].

### 3.1. Insulin: Mechanisms of Secretion, Action, and Their Involvement in Metabolic Alterations

Insulin is a 5.8 kDa peptide hormone that is released by pancreatic β-cells, whose main function is to regulate the concentration of glucose in the blood to promote the adequate storage of glucose in muscle and adipose tissue, as well as in the liver, which will consequently inhibit the hepatic glucose production. Other important functions of insulin are to promote cell division and growth, stimulate glycogen synthesis and inhibit its degradation in liver and muscle, inhibit lipolysis in adipose tissue, effects on gene expression, messenger ribonucleic acid (mRNA) turnover, ion uptake/retention, fluid and electrolyte balances, and control the critical energy functions associated with glucose metabolism, lipids, and proteins [26,27,28].

Under normal conditions, insulin synthesis begins with the production of preproinsulin via PI3K/p38MAPK/PKA/calmodulin kinase, followed by its transformation to proinsulin in the endoplasmic reticulum (ER), which is finally transported to the Golgi apparatus (GA) to form insulin, which will be secreted as soon as blood glucose levels increase. Glucose enters the pancreatic β-cells through a low-affinity glucose transporter (GLUT2); it is metabolized by glycolysis in the cytoplasm and by pyruvate carboxylase and pyruvate dehydrogenase in the mitochondria, thus generating an increase in the concentration of adenosine triphosphate (ATP). The change in the concentration of this molecule generates the consequent closure of ATP-sensitive potassium (K^+^) channels; this process generates a change in the action potential of the pancreatic cell that finally leads to the activation of L-type voltage-gated calcium (Ca^2+^) channels. The intracellular accumulation of Ca^2+^ generates the mobilization of insulin vesicles to the cell membrane and finally releases the insulin into the circulation (Figure 1) [16].

As mentioned, insulin release is stimulated by glucose concentrations, but it can also be modified by other factors such as free fatty acids, endocrine peptides, hormones (incretins, glucagon, etc.), and the autonomic nervous system. Thus, during diabetes, different metabolic conditions favor cellular stress in the ER and mitochondrial dysfunction, leading to protein misfolding, cell death, and insulin deficiency.

Once insulin is released into the circulatory system, it binds to its respective receptor tyrosine kinase in insulin-sensitive cells and, under normal conditions, two signaling cascades are triggered: the mitogenic pathway via the mitogen-activated protein kinases/Ras pathway; and the anabolic pathway via phosphatidylinositol-3-kinase (PI3K) stimulation [16]. Upon the binding of insulin to the extracellular α-subunits of insulin receptor (IR), the intrinsic tyrosine kinase activity of the β-subunits triggers autophosphorylation and the subsequent recruitment and phosphorylation of insulin receptor substrate 1 (IRS-1). This critical event bifurcates into two primary signaling cascades: the PI3K/AKT pathway and the mitogen-activated protein kinases (MAPK)/ERK pathway. In the metabolic pathway, phosphorylated IRS-1 activates phosphoinositide 3-kinase (PI3K), which catalyzes the conversion of phosphatidylinositol 4,5-biphosphate (PIP_2_) to phosphatidylinositol 3,4,5-triphosphate (PIP_3_) at the plasma membrane, thereby recruiting 3-phosphoinositide-dependent protein kinase 1 (PDK1) to phosphorylate and activate the serine/threonine kinase (AKT). Activated AKT serves as a master regulator of glucose homeostasis by promoting GLUT4 translocation for glucose uptake, stimulating glycogenesis through glycogen synthase activation, and enhancing lipid and protein synthesis via the mammalian Target of Rapamycin (mTOR) signaling axis. Concurrently, the mitogenic pathway is initiated through the recruitment of the growth factor receptor-bound protein 2 (Grb2-SOS) complex to IRS-1, which functions as a guanine nucleotide exchanged factor for the guanosine triphosphatase Ras (GTPase Ras). This triggers a sequential phosphorylation cascade of MAPK, involving Raf, MEK, and ERK. Once activated, ERK translocates to the nucleus to regulate transcription factors (TFs), thereby regulating gene expression, cell proliferation, and mitogenesis. Together, these coordinated pathways integrate the systemic anabolic and growth-promoting effects of insulin across hepatic, skeletal muscle, and adipose tissues (Figure 2) [29,30,31].

However, the same factors that affect the correct production of insulin, added to the increase in hepatic glucose production, alterations in lipid metabolism, and mutations in or post-translational modifications of the IR or downstream effector molecules, are a consequence of insulin resistance, that is, the inability of cells to recognize and respond to this hormone [24,25,26]. The most common alterations are a decreased number of receptors and their kinase activity, an increased phosphorylation state in serine/threonine (Ser/Thr) residues of key proteins such as the receptor and its substrate, decreased activity of PI3K and MAPK/Akt, and defects in the expression and function of the glucose transporter type 4 (GLUT4) [24,26,32].

### 3.2. Molecular Mechanisms Associated with the Pathophysiology of T2D

Defective insulin production and IR, caused by a combination of genetic and environmental factors, including obesity and an imbalance in adipokine production, are the initial steps in T2D development and the exacerbation of a cyclical state of hyperglycemia and chronic inflammation [33].

Inflammation is characterized by an imbalance between compensatory mechanisms and elevated levels of pro-inflammatory cytokines and chemokines. In the diabetic state, there is dysregulation of hepatic glucose production, since the action of insulin is generally reduced in the liver and peripheral tissues, causing elevated hepatic gluconeogenesis and glycogenolysis, a decrease in glucose uptake by peripheral tissues, and higher blood glucose levels [16].

T2D is often not easily diagnosed because hyperglycemia develops gradually and, in its early stages, is not severe enough to produce any of the classic symptoms of this disease. Patients with T2D may have insulin levels that appear normal or increased, and this can lead to the inability of this hormone to control hyperglycemia and lipid metabolism, although even cellular function may be normal [33,34].

Hyperglycemia, when not controlled, can generate a series of alterations such as an increase in free radicals, which are mostly reactive oxygen species (ROS), resulting from the decrease in antioxidant defense activity [35], among which are the enzymes superoxide dismutase (SOD), catalase (CAT), glutathione peroxidase (GPx), and glutathione reductase (GRed) [36,37,38]. This imbalance of the oxidant–antioxidant system produces oxidative damage in macromolecules, leading to oxidative alterations in deoxyribonucleic acid (DNA) during insulin resistance [39,40,41,42].

The presence of ROS has pathogenic implications, as it activates proinflammatory pathways involving redox-sensitive transcription factors, including nuclear factor kappa B (NF-κB), activator protein 1 (AP-1), early growth response 1 (EGR-1), and thioredoxin-binding protein; once activated, these proinflammatory factors in turn induce the presence of cytokines such as tumor necrosis factor-α (TNF-α), Interleukin 6 (IL-6), and C-reactive protein (CRP), which contribute to complications [42,43,44,45]. This decompensation affects the liver, muscle, adipose tissue, kidneys, and eyes, leading to pathologies: macrovascular complications (cardiovascular disease) and microvascular complications (retinopathy, nephropathy, and neuropathy) (Figure 3) [46], resulting in a general decrease in the quality of life in people with diabetes and in severe cases an increase in mortality [40,41,42].

One of the molecular mechanisms associated with the autooxidation of glucose is the activation of the enediol pathway, which consists of the conversion of α-D-glucose with an enediol radical, which, in the presence of transition metals, such as iron in the ferric state (Fe^3+^) or the manganese ion (Mn^+^), gives rise to the formation of the superoxide anion radical (O_2_^−^), which, when a dismutation reaction occurs, produces hydrogen peroxide (H_2_O_2_) and this in turn with the Fe^3+^ or Mn^+^ ion generates the hydroxyl radical (HO^−^); these free radicals induce the oxidation of proteins and lipids. Polyol, hexosamine, and protein kinase C (PKC) are end products of the glycosylation and glycolysis pathway that also induce oxidative stress due to hyperglycemia and, consequently, protein damage. Another process that occurs due to hyperglycemia is the reduction of glucose and the exposure time that leads to the reaction of the primary amino group of amino acids that are part of proteins through a peptide bond with the carbonyl group of reducing sugars forming a compound called Schiff’s base; all this, in the presence of ROS and nitrosative species, can cause proteins to mutate due to the reactions that occur with nucleic acids through lipid peroxidation (LPO) and, with it, damage to DNA [43,47,48]. With the overproduction of free radicals and the activation of NF-κB, a pro-inflammatory environment is induced in the cell with the consequent activation of vascular endothelial growth factor (VEGF) [43,49,50]. The activation of the above pathways leads to the development of micro- and macrovascular complications, such as diabetic retinopathy, nephropathy, and neuropathy, which are closely related to the years of evolution of the disease and glycemic control, due to the deficit in insulin functioning [43,47,50,51,52].

### 3.3. Drugs Used for the Treatment of T2D

While in T1D the only effective therapeutic strategy continues to be the administration of exogenous insulin, due to the absolute deficit of pancreatic insulin secretion, in T2D, there is a wide range of both pharmacological and non-pharmacological therapeutic options [53,54,55]. Current protocols for the treatment of T2D begin with non-pharmacological measures based on a balanced diet and body weight control. However, in many patients, these interventions are insufficient, and it is necessary to resort to the use of oral hypoglycemic agents aimed at improving glucose regulation and metabolism in the body [55,56]. Pharmacological alternatives are mainly aimed at correcting the pathophysiological defects characteristic of the disease, such as IR, pancreatic β-cell dysfunction, and alterations in glucose metabolism (Figure 4) [14,54,57].

In this context, metformin, representative of the biguanide group, continues to be the drug of first choice in most clinical guidelines, thanks to its efficacy, safety, and low cost [57,58]. When metformin monotherapy is insufficient, combination therapy represents the next alternative, incorporating different classes of oral drugs such as sulfonylureas and meglitinides, which act as insulin secretagogues; thiazolidinediones, which improve insulin sensitivity; α-glucosidase inhibitors, which reduce intestinal absorption of carbohydrates; incretin-based therapies, including glucagon-like peptide-1 (GLP-1) receptor agonists and dipeptidyl peptidase-IV (DPP-4) inhibitors; and sodium–glucose cotransporter type 2 (SGLT2) inhibitors [59,60]. Each of these pharmacological classes has distinct mechanisms of action, designed to correct one or more of the pathophysiological defects of T2D, and their selection depends on the individual clinical profile of each patient [27,61]. However, when treatment with oral agents is insufficient to achieve therapeutic objectives, exogenous insulin administration may be necessary, either on a baseline or intensive basis, to maintain adequate glycemic control and prevent microvascular and macrovascular complications [14,57,58].

Although all these options continue to be widely used in clinical practice, the safety and tolerability profile of each drug class should not be overlooked, as they are associated with different adverse effects that can range from mild discomfort to significant impairment of quality of life [16,62,63]. Sulfonylureas, for example, although still widely used, are associated with an increased risk of hypoglycemia, adverse cardiovascular events, and weight gain [64,65]; thiazolidinediones are associated with fluid retention, bone fractures, and, in the case of pioglitazone (PGZ), an increased risk of bladder cancer [66,67]; and DPP-4 inhibitors, although generally well tolerated, have shown in some studies a possible association with heart failure [68,69]. GLP-1 agonists, on the other hand, are associated with gastrointestinal adverse effects such as nausea and vomiting, as well as a rare risk of pancreatitis [70]. Finally, SGLT2 inhibitors may increase the risk of genitourinary infections and euglycemic ketoacidosis, especially in situations of metabolic stress or surgical procedures [71,72].

These limitations have driven the need to seek safer and more effective therapeutic alternatives, with greater potency and selectivity and a better cost–benefit ratio, based on new structural prototypes and innovative approaches in medicinal chemistry. In this sense, GLP-1 agonists and SGLT2 inhibitors have gained special relevance in recent years, not only because of their efficacy in reducing glycated hemoglobin, but also because of the additional benefits demonstrated in cardiovascular and renal protection [14,60,71,73,74,75]. However, beyond these pharmacological classes, new molecular pathways and targets are continuously being explored to expand the available therapeutic arsenal.

## 4. Advances in Research into Antidiabetic Treatments

The search for new therapeutic strategies for diabetes and associated metabolic imbalances has intensified in recent years, driven by a better understanding of the underlying molecular mechanisms and the development of multitarget approaches. While there is still a great deal of interest in optimizing conventional targets, several studies also explore metabolic enzymes and transcription factors involved in energy regulation and insulin sensitivity.

A prominent example is glucokinase (GCK), recognized as a key regulator of glycemic homeostasis. Genetic variants in the GCK gene have been closely associated with the risk of developing diabetes, making this enzyme an attractive target for pharmacological research [76]. In parallel, the activation of AMP-activated protein kinase (AMPK) by compounds such as PXL770 and PBI4050 is being investigated as a strategy to improve energy regulation and insulin sensitivity, reinforcing the importance of cellular energy sensors as a therapeutic target [60,76]. Other advances include the study of sirtuin-activating compounds, particularly SIRT1, an enzyme involved in lipid metabolism, inflammation, and insulin sensitivity [76,77].

Other emerging targets for the treatment of T2D include enzymes and proteins involved in metabolic regulation and insulin signaling. These include 11β-hydroxysteroid dehydrogenase (11β-HSD), which modulates the conversion of cortisone to cortisol and is associated with IR and obesity; glutamine fructose-6-phosphate aminotransferase (GFAT), a key regulator of the hexosamine pathway and linked to insulin resistance; and protein tyrosine phosphatase 1B (PTP1B), a negative regulator of insulin and leptin signaling. Genes and proteins such as SLC16A11, linked to lipid metabolism and the genetic risk of T2D, CCN3/NOV, associated with obesity, inflammation, and IR, and the transcription factor FOXO1, a mediator of apoptosis and β-cell dysfunction, have also been identified. Other promising targets include the FFAR2/FFAR3 fatty acid receptors, involved in modulating insulin secretion, and epoxyeicosatrienoic acids (EETs), metabolites of arachidonic acid with anti-inflammatory and insulin-sensitizing properties. Together, these findings expand the therapeutic landscape beyond classical targets and open new opportunities for the development of innovative antidiabetic agents [77,78].

However, clinical experience has shown that single-target therapies are often insufficient to address the complexity of multifactorial diseases such as diabetes. Consequently, the need for combination therapies has been highlighted, although these entail limitations related to pharmacokinetics, toxicity, or treatment adherence [71]. As an alternative, in the last decade, the design of multitarget ligands has emerged to simultaneously modulate, designed to simultaneously modulate two or more therapeutic targets and thus improve long-term efficacy and safety [79,80,81,82,83,84].

One of the most widely used strategies in this field is to take advantage of different pharmacophores, active on individual targets, and integrate them into a single hybrid molecule. The success of this approach depends on the pharmacophoric elements maintaining their ability to interact with specific binding sites, simultaneously producing the desired effects. The partial superposition or fusion of pharmacophores is often used to optimize the overall activity of the molecule. However, this process requires careful structural balancing to modulate the relative potency towards each target and achieve an optimal pharmacological profile [79,80,81,82,83,84].

## 5. Antidiabetic Application of Benzothiazole-Based Compounds

Benzothiazole is a bicyclic heterocyclic molecule containing a benzene ring fused with a thiazole ring. It is relatively stable due to its aromaticity, even though it is a weak base and a heterocycle with reactive sites that allow it to be functionalized and multiple substituents to be added, giving a wide variety of biological and pharmacological activities, mainly due to the modification of the structure at the C-2 position [85].

These molecular bases have been found in multiple natural compounds, in addition to being studied as antitumor, antimicrobial, antidiabetic, anti-inflammatory, anticonvulsant, antiviral, antioxidant, antituberculous, antimalarial, antiasthmatic, anthelmintic, diuretic, analgesic, and photosensitizing, among others (Figure 5). These molecules have nitrogen and sulfur atoms that act as electron donor and acceptor groups, conferring important pharmacological effects due to their interaction with biological targets, such as those addressed in this paper, involved in diabetes treatment [85,86].

Despite extensive research into these novel pharmacotherapies, clinical trial databases indicate that benzothiazole-derived molecules have not yet been formally evaluated as agents for diabetes or obesity; however, a substantial body of preclinical evidence, encompassing in silico, in vitro, and even in vivo or ex vivo studies, supports their evaluation as agents for the management of metabolic diseases.

### 5.1. In Vivo Evaluation

Interest in benzothiazoles in chemical and pharmacological research was consolidated during the 1940s and 1950s, when their applications in the biomedical field began to become evident. Since then, this heterocyclic nucleus has attracted growing interest as a versatile structural scaffold, particularly in the design of new molecules with antidiabetic potential, both due to its ability to modulate key enzymes in metabolic processes and its participation in pathways associated with the control of hyperglycemia. These evaluations have been performed using many methodologies, including and excluding an explanation of their possible action mechanism.

In 2005, Pattan et al. evaluated the acute hypoglycemic activity of a series of compounds derived from 2-amino [5’(4-sulphonylbenzylidine)-2,4-thiazolidinenone]-7-chloro-6-flurobenzothiazole, at a 36 mg/kg single dose (Figure 6A), and determined their potential hypoglycemic activity in an acute study on alloxan-induced diabetic rats. All of them decreased glycemic values 1 h after treatment, and this effect remained up to 6 h, reaching levels comparable to those of healthy controls [87].

In the study by Mariappan et al. (2012), a series of benzothiazole derivatives, identified as N-(6-chlorobenzothiazol-2-yl)-2-(amino-substituted)acetamides 3(a–j), were synthesized and evaluated for their antidiabetic activity in rats with streptozotocin-induced diabetes (STZ). As shown in Figure 6B, the series shares a common 6-chlorobenzothiazolacetamide core and varies only in the amino substituent –NR_1_R_2_, allowing for a comparison of the effect of different functional groups on the hypoglycemic response. The results indicated that all the evaluated compounds produced a reduction in hyperglycemia after the 10-day experimental period. Among them, compound 3d, which contains a morpholino group, exhibited the most significant activity within the series at an oral dose of 100 mg/kg. Toxicity was also assessed; the compounds were administered to the animals, and they were kept under observation for signs of neurological toxicity, such as increased or decreased motor activity, tearing, sedation, seizures, or tremors. As no signs of toxicity were observed and with an overall mortality rate of zero percent, the cut-off value was set at 100 mg/kg as LD_50_ [88].

On the other hand, in 2013, Meltzer et al. obtained a series of compounds from 2-chloro-5-((Z)-((E)-5-((5-(4,5-dimethyl-2-nitrophenyl)furan-2-yl)methylene)-4-oxothiazolidin-2-ylidene)amino)benzoic acid, which increased the glucose uptake rate by activating adenosine 5’-monophosphate-activated protein kinase (AMPK), which promotes the translocation of GLUT4 receptors to the skeletal muscle cell membrane. They demonstrated through in silico, in vitro, and in vivo studies that compound 34 (2-(benzo[d]thiazol-2-ylmethylthio)-6-ethoxybenzo[d]thiazol), shown in Figure 6C, yielded the best results at a concentration of 25 μM; since it phosphorylates AMPK and subsequent enzymes without interfering with the insulin transduction pathway, thus reducing hyperglycemia, it suggests that compounds containing benzothiazole in their chemical structure are crucial to achieving the effect on GLUT4 receptor translocation [89].

In response to the increased interest in five-membered heterocycles in drug design, Kapoor and Khare (2016) synthesized a series of hybrid benzothiazole derivatives with thiazolidinedione and rhodanine cores, designed from the PGZ pharmacophore. As shown in Figure 6D, the overall structure retains a lipophilic benzothiazole region, an acetamide-ether linkage, and a variable heterocyclic core, where R_1_ = O corresponds to thiazolidinedione derivatives and R_1_ = S to rhodanine derivatives. The modification at R_2_ allowed the exploration of different amino substituents, with compound 3, identified as MB01, being particularly noteworthy. This piperazine-containing compound exhibited the highest antihyperglycemic activity of the series, achieving a 46.46% reduction in glucose levels at 6 h in rats with fructose-induced diabetes. Although its effect was less than that of PGZ, these results suggest that combining benzothiazolidinedione with piperazine substitution may be a promising strategy for the design of new antidiabetic agents [90].

In Kumar et al. (2016), a series of compounds containing oxadiazole linked to benzothiazole (6a–6l) was proposed. These compounds were administered at a dose of 350 mg/kg for 14 consecutive days. The results showed a reduction in blood glucose of up to 66.84%, similar to the action of glibenclamide at a dose of 10 mg/kg. Most of the compounds were effective, with compound 6f (Figure 6E) showing the best activity at 350 mg/kg. However, the mechanism of action of these compounds is not specified. It can be suggested that the combination with benzothiazole-type substituents potentiates the antidiabetic activity [91].

A year later, Kumar et al. (2017) synthesized and evaluated a series of 7(a–l) benzothiazol-thiazolidinedione derivatives, whose general structure is shown in Figure 6F. These compounds incorporate a benzothiazole core linked by a thioacetamide bond to a thiazolidinedione ring, with variations at R_1_, R_2_, and R_3_. In the alloxan-induced diabetes model, most of the derivatives showed a blood glucose reduction of approximately 40–60% at a dose of 350 mg/kg, compared to glibenclamide, which achieved a reduction of 65.8%. Compound 7d was the most active in the series, with a reduction of 63.44%, suggesting that the combination of a methyl group on the benzothiazole and an *m*-nitrophenyl substituent promotes antidiabetic activity [92].

Based on Kumar’s experimental background, Thube et al. (2021) synthesized a series of 2-substituted benzothiazole 3(a–j) derivatives, whose general structure is shown in 6G, incorporating electron-withdrawing substituents such as bromine, chlorine, and nitro groups. These compounds were evaluated as antidiabetic agents in rats with alloxan-induced diabetes, showing favorable results in a diabetic control. Compound 3d was the most active in the series, reducing glucose from 363.3 to 102.8 mg/dL by day 12, equivalent to an approximate decrease of 71.7%, comparable to PGZ, which achieved a 72.0% reduction. Furthermore, several derivatives also decreased cholesterol and triglyceride levels, suggesting a broader metabolic effect. These results highlight the importance of incorporating bromine and chlorine as substituents in the benzothiazole core and the acetanilide portion to promote antidiabetic activity [93].

### 5.2. α-Amylase and α-Glucosidase as Antidiabetic Targets

α-Amylase and α-glucosidase are carbohydrate digestive enzymes primarily located in the brush border of the intestine and responsible for the breakdown of polysaccharides into monosaccharides [60]. Therefore, these enzymes are essential in glucose metabolism. α-Amylase hydrolyzes internal α(1–4) glycosidic bonds in polysaccharides such as amylose and amylopectin, while α-glucosidase subsequently degrades oligosaccharides and polysaccharides into glucose, which, when absorbed, enters the bloodstream. Therefore, inhibiting α-amylase and α-glucosidase enzymes can suppress carbohydrate digestion, delay glucose uptake, and consequently, reduce and regulate postprandial blood sugar levels. Although drugs such as acarbose, voglibose, and miglitol inhibit α-glucosidase and α-amylase, slowing the action of both enzymes, reducing starch hydrolysis, and having a beneficial effect on glycemic index management in diabetic patients in practice, they produce undesired side effects such as bloating, abdominal discomfort, diarrhea, and flatulence [94,95]. These enzymes do not belong to the same family; however, both carry out acid–base hydrolysis reactions and have a catalytic site formed by an aspartic amino acid residue that acts as a nucleophile, another that stabilizes the transition state of the substrate, and a glutamic residue that acts as an electron donor [96]. In addition, the active site environment includes other residues that mediate hydrophilic and hydrophobic interactions with substrates and inhibitors, as well as established hydrogen bonds with His305, Gly306, His201 (α-amylase), and Lys155, Glu276 (α-glucosidase) [97].

#### Recent Research on Benzothiazole Derivatives as α-Amylase and α-Glucosidase Inhibitors

Puranik et al. synthesized 15 derivatives from benzothiazole (**3a**–**3p**) (Figure 7A), and they evaluated their potential as inhibitors of α-glucosidase, α-amylase, and advanced glycation end products (AGEs). As an indicator of diabetes control, in vitro non-enzymatic hemoglobin glycosylation assays and molecular coupling studies were performed to determine unrelated interactions. Compound **3l** showed the best activity with low IC_50_ values of 0.31, 0.98, 0.59, and 0.19 mM in α-amylase, α-glucosidase, non-enzymatic glycosylation of hemoglobin, and AGE inhibition assays, respectively. Those values were more promising than acarbose, and it has a Ki of 0.39 and 1.5 mM for α-amylase and α-glucosidase, respectively. In addition, they showed that molecule **3l** binds in the catalytic active site pocket of α-glucosidase and α-amylase [94].

Gong et al. synthesized 19 compounds with a triazole-based structure fused to a benzothiazole (6a–6s) (Figure 7B). In vitro inhibitory activity against α-glucosidase varied with IC_50_ values between 20.7 and 61.1 µM, being better than the standard acarbose (IC_50_ = 817.38 µM). The most active compound was **6s** (IC_50_ = 20.7 µM) bearing a chlorine group at the 5-position of the benzothiazole ring and a tert-butyl group at the para position of the phenyl ring. Molecular docking studies were performed based on the binding modes and interactions; they determined that molecules with a chlorine (Cl), fluorine (F), and bromine (Br) halogen group on the phenyl group increased their inhibitory activity through an interaction with the enzyme of α-glucosidase pocket (12).

In 2018, Shah et al. reported the synthesis of twenty-five small molecules derived from 5-chloro-2-aryl benzo[d]thiazole (1–25) (Figure 7C), whose inhibitory activity against α-glucosidase was assessed in vitro. The most active compounds 4, 9, and 10 showed IC_50_ values in the range of 22.1 ± 0.9 to 25.6 ± 1.5 µM. While compounds 2, 5, 11, and 19 showed IC_50_ values within the range of 40.2 ± 0.5 to 60.9 ± 2.0 µM and were compared with acarbose (standard) (IC_50_ = 840 ± 1.73 µM), compounds 1 and 3 were also found to be good inhibitors with IC_50_ values of 136.2 ± 5.7 and 104.8 ± 9.9 µM, respectively. Kinetic studies indicated compounds 2, 3, 10, 19, and 25 to be non-competitive, while 1, 5, 9, and 11 were competitive inhibitors of α-glucosidase enzyme. All the active compounds (1–5, 9–11, and 19) were also found to be non-cytotoxic, in comparison to the standard drug doxorubicin (IC_50_ = 0.80 ± 0.12 µM) in an MTT assay. In silico studies of active compounds with enzyme-binding sites were also employed to validate successful interactions [95].

On the other hand, Mor et al. synthesized and evaluated the antibacterial, antifungal, and α-amylase- and α-glucosidase-inhibitory activity of a series of benzothiazolyl hydrazones (3a–3o) (Figure 7D) In in vitro assays, they demonstrated that hydrazone 3e (IC_50_ = 23.07 μg/mL) and 3n (IC_50_ = 22.57 μg/mL) showed better activity on α-amilase, whereas compounds 3j (IC_50_ = 2.06 μg/mL) had better inhibition of α-glucosidase. It was revealed that its inhibitory potential depends primarily on the substituents on the phenyl ring, not on those on the benzothiazole. However, the absence of substituents on the benzothiazole decreased its activity against α-glucosidase [98].

Gollapalli et al. synthesized a series of 23 oxadiazoles with a benzothiazole nucleus (1–23) to obtain a new inhibitor of the α-glucosidase (Figure 7E). All the molecules presented a higher inhibitory activity (IC_50_ values ranging 0.5 ± 0.01–30.90 ± 0.70 μM) than the reference drug acarbose (IC_50_ 866.30 μM). They found that the molecules with the highest biological activity are those that have a dihydroxy and monohydroxy substituent in the phenyl ring of the base structure, where the position also influences the potential. They also performed molecular coupling studies on the active site of the enzyme, obtaining affinity energies of benzothiazole analogues. Likewise, in the binding mode and ligand–receptor interactions, they found that hydroxy groups play an important role in the formation of hydrogen bonds [99].

Mor et al. synthesized a series of benzothiazolyl hydrazones (indeno [1,2-c]pyrazol-4(1H)-ones) (4a–4o) (Figure 7F) and evaluated its antibacterial and antifungal activity. On the other hand, the activity of derivatives on α-amylase and α-glucosidase was evaluated with in vitro assays. The molecules 4e (IC_50_ 6.71 μg/mL) and 4i (IC_50_ 8.18 μg/mL) showed better activity based on α-glucosidase compared to the reference drug acarbose with an IC_50_ of 9.35 μg/mL, whereas compounds 4i (IC_50_ 11.90 μg/mL) and 4o (IC_50_ 12.83 μg/mL) had better inhibition of α-amylase compared to acarbose with an IC_50_ of 22.87 μg/mL (Figure 7F). Hence, molecular docking was performed using these compounds, respectively, with each of the enzymes with important interactions [100].

Building on previous studies demonstrating that 2-mercaptobenzothiazole derivatives exhibited antiglycation activity without cytotoxic effects, Ullah et al. synthesized a series of 33 compounds [1,2,3,4,5,6,7,8,9,10,11,12,13,14,15,16,17,18,19,20,21,22,23,24,25,26,27,28,29,30,31,32,33] (Figure 7G) and evaluated their ability to inhibit the α-glucosidase enzyme. Several derivatives (2–4, 6–7, 9–26, 28 and 30) exhibited significantly greater inhibitory activity (IC_50_ = 31.21–208.63 µM) than the standard drug acarbose (IC_50_ = 875.75 ± 2.08 µM). Furthermore, most of these compounds had previously demonstrated potent antiglycation activity, reinforcing their potential as dual agents, with an effect on both inhibiting carbohydrate digestion and preventing complications associated with glucotoxic stress. This suggests that 2-mercaptobenzothiazole derivatives represent a promising structural platform for the development of novel antidiabetic drugs with complementary mechanisms of action [101].

Vijayan et al. synthesized and evaluated a set of five benzothiazole derivatives bridged with indenedione through a methylenehydrazone linker (BZT1–BZT5). (Figure 7H), aiming to explore their antidiabetic potential as inhibitors of α-glucosidase and *α*-amylase. The results obtained from the glucose uptake assay in L6 myoblast cells showed that BZT1 and BZT4 exhibited high inhibition of glucose uptake (89.07 and 81.54% respectively) compared with PGZ (83.68%). But only the derivative BZT4 (IC_50_ 48.8 μg/mL) showed inhibition against α-glucosidase compared with acarbose (IC_50_ 39.1 μg/mL), while in α-amylase inhibition, BZT1 (IC_50_ 51.46 μg/mL) was close to the reference drug acarbose (IC_50_ 28.47 μg/mL). The authors suggested that such derivatives could represent a promising scaffold for the design of multifunctional antidiabetic agents based on docking studies [102].

In 2022, Srinivasa et al. synthesized ten benzothiazole-rhodanine derivatives as potent *α*-amylase and α-glucosidase inhibitors (A1–A10) (Figure 7I). Rhodanine-fused aminobenzothiazole derivatives exhibited remarkable inhibitory activity against α-amylase and α-glucosidase, mainly attributed to the presence of electron-donating groups at specific positions of the phenolic ring. In an α-amylase assay, compounds A5 (IC_50_ 24.22 ± 1.65 μM) and A9 (IC_50_ 24.64 ± 1.88 μM) showed comparable efficacy to the standard acarbose (IC_50_ 24.35 ± 1.44 μM), while in an α-glucosidase assay, compounds A6 (IC_50_ = 26.37 ± 1.76 μM) and A7 (IC_50_ = 24.74 ± 1.63 μM) showed potent activity and compounds A9 (IC_50_ = 24.61 ± 1.76 μM) and A10 (IC_50_ = 26.22 ± 0.91 μM) demonstrated moderate efficacy when compared to the standard (23.73 ± 1.22 μM). They studied the ligands’ in silico properties and interactions with both enzymes. In contrast, electron-withdrawing substituents such as Cl, Br, or NO_2_ reduced the inhibitory activity, placing these compounds in the moderate to weak efficacy range. Pharmacophoric studies identified three hydrogen acceptors and two aromatic hydrophobic groups, consistent with molecular docking. These results consolidate aminobenzothiazole-rhodanine derivatives as promising candidates for enzyme inhibition [103].

Then, Patil et al. synthesized 35 derivatives (GB1–GB35) (7J); the compounds GB24, GB21, GB16, GB17, and GB25 exhibited very significant inhibitory activity, with an IC_50_ of 29.91 ± 0.82, 36.52 ± 0.49, 39.19 ± 1.09, 41.35 ± 1.37, and 60.47 ± 1.49 μM, respectively, demonstrating higher potency than acarbose (IC_50_ 67.06 μM) (Figure 7J). A molecular docking study was performed confirming the binding interactions with the target enzyme based on the findings; they can predict that these compounds could be taken further for the development of new antidiabetic agents supporting their role as potent α-glucosidase inhibitors [104].

It has been recognized that the type of biological activity of benzothiazoles is mainly due to the C-2 substituents; however, Khan et al. designed 15 molecules via functionalization of the other carbon atoms with thiazolidinone derivatives [1,2,3,4,5,6,7,8,9,10,11,12,13,14,15] (Figure 7K). They found that all compounds showed inhibitory activity, with IC_50_ values in the range of 2.10–37.50 µM for *α*-amylase and 3.20–39.40 µM for *α*-glucosidase, highlighting derivatives 4 (2.40 ± 0.70 and 3.50 ± 0.70 µM), **5** (2.30 ± 0.05 and 4.80 ± 0.10 µM), and **6** (2.10 ± 0.70 and 3.20 ± 0.70 µM) that presented significantly greater potency than the standard drug acarbose (IC_50_ = 9.10 and 10.70 µM, respectively). A clear structure–activity relationship was established where the presence and position of substituents such as fluorine and hydroxyl groups enhanced the inhibitory activity, while electron-withdrawing groups tended to decrease it. Molecular docking studies showed that the most active compounds exhibited stable binding modes and favorable affinity energies in the active site of both enzymes [76]. In a subsequent study, Khan et al. advanced their research by synthesizing S-substituted benzothiazole derivatives incorporating thiazole rings and bis-Schiff bases [1,2,3,4,5,6,7,8,9,10,11,12,13,14,15,16,17,18,19] (Figure 7L) to evaluate their inhibitory activity against *α*-glucosidase and *α*-amylase enzymes. The compounds showed remarkable inhibitory potency, with IC_50_ values ranging from 2.10 ± 0.78 to 21.76 ± 0.19 µM for α-glucosidase and from 1.40 ± 0.59 to 19.84 ± 2.10 µM for α-amylase, surpassing acarbose as the reference drug in several cases (IC5_50_ = 6.45 ± 1.84 µM for α-glucosidase; IC_50_ = 4.30 ± 0.18 µM for α-amylase). Compounds **1** (IC_50_ 3.18 ± 0.72 to and 2.30 ± 1.80) and 4 (IC_50_ 1.40 ± 0.59 and 2.10 ± 0.78) to α-amylase and α-glucosidase, respectively.

As in their previous work, a structure–activity relationship (SAR) analysis was established, which, in this case, allowed for more solid conclusions: substituents in the *para* position significantly increased their efficacy, while those in the *ortho* position reduced it. In particular, the fluorine and trifluoromethyl groups demonstrated greater inhibitory potential, with analogue 4 (with a *para–CF*_3_ group) standing out, exhibiting one of the best activities in the series. Molecular docking studies confirmed the affinity of the ligands for the active site of the enzymes, where compound 19d reached ∆G values of –9.03 and –8.28 kcal/mol. Although it did not interact directly with the catalytic triads, its high activity is attributed to its ability to establish stabilizing interactions mediated by halogen atoms [105].

For its part, Sevimli E. et al. synthesized seven compounds by reacting 6-substituted-2-aminobenzothiazole compounds with 2,4,6-trihydroxybenzaldehyde (3a–3g) (Figure 7M) and then subjected them to an in silico and in vitro evaluation based on α-glucosidase. The IC_50_ was calculated to be 24.05 ± 2.28 and 18.51 ± 1.19 μM for the best compounds 20f and 20g**,** respectively. This inhibition was determined to be non-competitive, which was supported by molecular docking, which demonstrated the importance of phloroglucinol hydroxyls interacting with key α-glycosidase residues and aspartate residues of the catalytic triad. In this case, the presence of a fluorine atom does not improve the inhibitory activity (IC_50_ 57.20 mM), corroborating the importance of the position of the substituents [106].

On the other hand, Pilotzi-Xahuentitla et al. synthesized the compound *N*-(benzothiazole-2-yl)-4-(5-chlorobenzoxazole-2-yl) aminobutanamide (5) (Figure 7N). This was subsequently subjected to in silico evaluation via molecular docking studies with a 3-TOP protein, a human maltase-glucoamylase (glucagon 1,4-a-glucosylase). It showed a ∆G of −8.4 Kcal/mol. In addition, through this evaluation, the importance of benzothiazole can be highlighted, since it has a better interaction with the active site than benzoxazole [107].

Hussain et al. developed a novel series of derivatives integrating a fluor-substituted imidazopyridine structure with a thiadiazole moiety retaining the S-substituted benzothiazole structure [1,2,3,4,5,6,7,8,9,10,11,12,13,14,15,16,17,18] (Figure 7O), in order to evaluate them as inhibitors of carbohydrate-digesting enzymes in vitro. The molecules have shown promising results as dual inhibitors in the ranges of IC_50_ = 12.20–46.90 µM (α-amylase) and 12.50–48.60 µM (α-glucosidase), outperforming acarbose (32.40 ± 2.30 and 35.70 ± 2.70 μM, respectively). Specifically, the compounds that showed significantly better inhibition were 13, 10, 9, 7, and 3. Furthermore, SAR analysis indicates that, to a lesser extent, chlorine also favored activity, while methyl tended to be the weakest within the compared series. Molecular dockings against both targets were consistent with experimental results. Finally, 100 ns molecular dynamic simulations for the protein–ligand complexes yielded RMSD values below ~3 Å, supporting the observed conformational stability and affinity [108].

### 5.3. Dipeptidyl Peptidase 4 (DPP-4)

DPP-4 is a key serine protease in the pathophysiology of T2D, as it degrades GLP-1, an essential incretin in the stimulation of insulin biosynthesis and release, the inhibition of glucagon secretion, and the regulation of pancreatic β-cell mass. Its inhibition prolongs the half-life of active GLP-1 and potentiates its effects, constituting an effective therapeutic strategy that avoids adverse effects such as hypoglycemia or β-cell depletion. Structurally, DPP-4 consists of a β-propeller domain associated with an α/β-hydrolase domain, at the interface of which is the catalytic triad consisting of Ser630, Asp708, and His740. The active site also includes an oxyanion hole (Tyr547 and Ser631), a hydrophobic S1 pocket (Tyr631, Val656, Trp659, Tyr662, Tyr666, Val711), and a conserved recognition motif in the acidic loop Glu205/Glu206. Other residues such as Arg125, Val207, Phe357, Arg358, and Asn710 are also involved in ligand binding and catalytic mechanisms. This complex architecture determines the binding selectivity of the inhibitors, in which hydrophobic interactions, hydrogen bonds, and salt contacts with Glu205/Glu206 and Tyr662 are essential for the stabilization of the complex. In this context, DPP-4 inhibitors represent a consolidated approach to modulate the incretin pathway and improve glycemic control in T2D [109,110,111].

#### Recent Research of Benzothiazole Derivatives as DPP-4 Inhibitors

In Ferraris et al. (2004), two families of compounds were studied: ketopyrrolidines and ketoazetidines (5 a–h, 7 a–d) (Figure 8A1,A2). Once synthesized, their activity as DPP-4 inhibitors was evaluated through assays complemented by stability studies and ex vivo tests in rats. Among the results, the derivatives containing the benzothiazole group stood out for their high potency. In the series of ketopyrrolidines, compound 5b showed an IC_50_ of 30 nM, making it one of the most active, comparable to the 2-thiazole derivative. Similarly, in the series of ketoazetidines, compound 7b exhibited an IC_50_ of 80 nM, thus maintaining high inhibitory activity. These results demonstrated that the benzothiazole group is optimal for the S1’y S2 sites of the enzyme and confers great potency to both ketopyrrolidines and ketoazetidines. The discussion highlights that the presence of nitrogen on the aromatic ring is essential for interactions with the Arg125 residue of DPP-4, which explains the superiority of benzothiazole derivatives over phenyl, methyl, or other heteroaromatic rings such as thiophene, which were found to be practically inactive. Furthermore, stability studies indicated that benzothiazole-containing ketoazetidines were more stable than equivalent ketopyrrolidines, retaining a significant proportion of the active form for several hours [112].

Later on, Nitta et al. designed, synthesized, and evaluated a novel series of 3-amino-N-(4-aryl-1,1-dioxothian-4-yl)butanamides and 3-amino-N-(4-aryltetrahydropyran-4-yl)butanamides as DPP-4 inhibitors, 15 of which contained a benzothiazole moiety (12 j–x) (Figure 8B). Derivatives incorporating the 6-substituted benzothiazole group showed highly potent activity. Compounds 12v and 12x were found to be the most potent inhibitors, with IC_50_ values in the subnanomolar range (0.64 and 0.79 nM, respectively). These findings did not align with those previously reported in the literature, so the authors concluded that they provide new insights into the SAR in the inhibition of this enzyme. Of those evaluated, compound 12u showed the greatest in vitro stability against CYP metabolism in humans (CLint = 0.0219 mL/min/mg). Furthermore, after oral administration to rats, this compound maintained its metabolic stability and exerted a potent dose-dependent inhibitory effect on plasma DPP-4 activity (ID_50_ = 2.7 mg/kg). Finally, in an oral glucose tolerance test in ICR mice, oral administration of 12u at 30 mg/kg significantly reduced blood glucose fluctuations [113].

Finally, in a 2015 review, Kamal et al. found that the benzothiazole-containing molecule [58] (Figure 8C) can inhibit DPP-1 with an IC50 of 0.74 µM. They described DPP-1 inhibition as useful in the treatment of diabetes (Kamal, 2015). However, DPP-1 is associated with neutrophil-mediated inflammatory diseases and not with diabetes mellitus, so this information needs to be corroborated. It is worth noting that benzothiazole-core compounds also inhibit other dipeptidyl peptidase isoforms, highlighting the importance of this heterocycle with multiple biological activities [114].

### 5.4. Peroxisome Proliferator-Activated Receptor (PPAR)

PPARs belong to the nuclear receptor superfamily and act as ligand-inducible transcription factors. There are three distinct and highly homologous subtypes: PPARs, alpha (PPAR-α), beta/delta (PPAR-β/δ), and gamma (PPAR-γ). The latter is the most studied, as it regulates the expression of various genes involved in carbohydrate energy metabolism and acts as a modulator of inflammation and adipogenesis [49,115]. Therefore, selective agonism of these receptors may improve metabolic abnormalities associated with T2D, such as hyperglycemia, hyperlipidemia, insulin resistance, and other cardiovascular risk factors [27,116]. PPAR-γ activators include prostanoids, fatty acids, TZDs, and N-(2-benzoylphenyl) tyrosine analogues, which act as insulin sensitizers [116].

The ligand-binding pocket (LBP) of PPAR-γ is approximately 1200 Å^3^ in size and is located at the center of the ligand-binding domain (LBD). This LBP is described as a large Y- or T-shaped cavity composed of three branches. Branch I, predominantly polar, consists of helices H3, H5, H11, and H12; branch II, apolar, includes helices H2′, H3, H6, and H7, as well as the β-sheet region; and branch III, which combines polar and apolar regions, encompasses helices H2, H3, and H5 [27,49]. Within LBP, residues reported as part of the ligand-binding site include [27,115].

There are key residues that induce receptor activation depending on the type of agonist action, whether a full or partial agonist. In this case, the molecules could behave as partial agonists, interacting with important residues such as Cys285, Arg288, and Ser289 of helix 3, promoting a conformational change in helix 12 without directly interacting with it [117].

#### Recent Research on Benzothiazole Derivatives as PPAR-γ Agonists

Li et al. (2008) identified a novel synthetic PPAR-γ ligand (2-chloro-N-(3-chloro-4-(5-chloro-benzo[d]thiazol-2-ylthio)phenyl)-4-(trifluoromethyl)benzenesul-fonamide (T2384) (Figure 9A), which was evaluated for its biological activity in vitro in HEK293 cells in the absence or presence of 0.3 of rosiglitazone; T2384 was able to efficiently antagonize the ability of rosiglitazone to convert the preadipocytes into adipocytes. In in vivo studies in KKAy diabetic mice systemically treated with T2384, insulin sensitivity was rapidly improved in the absence of weight gain, hemodilution, and anemia, characteristics of rosiglitazone (RSG) treatment, while upon co-administered with rosiglitazone, T2384 was able to antagonize the side effects induced by rosiglitazone treatment alone, while maintaining effects on the normoglucemic level. In silico in X-ray crystallography studies revealed that T2384 can adopt two distinct binding modes, designated “U” and “S,” interacting with the PPAR ligand-binding cavity primarily through hydrophobic contacts, unlike full agonists. The different binding modes occupied by T2384 induced distinct patterns of coregulatory protein interaction with PPAR-γ [118].

Kharbanda et al. suggest that some sulfonylurea can improve insulin sensitization via PPAR-γ agonism, in addition to their known effect on insulin secretion. They synthesized 28 sulfonylurea/sulfonylthiourea compounds with a benzothiazole moiety (7a–7l, 8a–8l) (Figure 9B). The in vitro antidiabetic activity of the best compounds (7c, 7d, 7i–7l, 8c, 8d, 8g, and 8h) was evaluated via the OGTT in PPAR-γ transactivation assays in the 3T3 cell line, which showed potent antidiabetic activity greater than 50% compared with standard drugs. In in vivo studies, oral administration for 15 days of treatment reduces the plasma glucose concentration in a model of T2D in rats treated with STZ, finding that 7c, 7d, 8c, and 8d compounds significantly decreased plasma glucose levels compared to glibenclamide and rosiglitazone in rats. In molecular docking studies on the PPAR-γ protein, molecules with a better binding energy, around ΔG = −10.06 Kcal/mol, compared to rosiglitazone (−5.72 Kcal/mol), were found, determining that molecule 7j similarly increases gene expression in response to rosiglitazone. It was concluded that the combination of a benzothiazole nucleus with a sulfonylurea leads to the design of molecules with biological activity and pharmacological potential [119].

In another paper, Kharbanda et al. analyzed 20 benzothiazole derivatives (5a–5j) and (6a–6j) PPAR-γ agonism (Figure 9C). In vitro transactivation assays of PPAR-γ activation mediated by the derivatives (5b, 6a–6h) were shown to increase PPAR-γ-target gene expression, -fold, outperforming piperine (0.71), although with lower potency than rosiglitazone. The compounds were subsequently tested using in vivo models using OGTT and in STZ-induced diabetic rats; active derivatives (5b, 6a–6h) showed more antidiabetic potential than rosiglitazone, and these compounds were also studied for their effect on body weight, lipid peroxidation (LPO), and hepatotoxicity, showing a more favorable profile than the standard. In hepatic safety, the animals treated with 6c and 6d showed a normal histological pattern in liver tissue, unlike diabetic controls that presented with degeneration and fatty changes. Furthermore, both derivatives significantly reduced LPO, with minimal increases in LPO (11.29% and 8.87%, respectively), compared to the 38.70% increase observed with rosiglitazone, suggesting a lower toxic potential. Among all the derivatives, 6c and 6d were the most active, normalizing glucose levels without causing excessive weight gain. The molecular docking showed ∆G values around −7.00 Kcal/mol and adequate accommodation of the ligands in the receptor pocket [120].

On the other hand, in 2017, Pirat et al. designed a series of *α*-ethoxyphenylpropionic acid derivatives containing benzothiazol-2-one and incorporating resveratrol or butein scaffolds (13a, 13b, 13e, 13f, 14a–14d, 15a, 15b, 18a, 18b, 18e, 18f, 19a, 19b) (Figure 9D). In in vitro studies, the stipene compounds acted as full agonists of PPAR-γ. Compound 4d showed the best full agonist activity of the PPAR-*γ* receptor (Emax = 98%, EC50 = 200 nM), enzymatic activation of SIRT1 (+128%), and inhibition of SGK1 expression (−57%), minimizing side effects such as fluid retention and weight gain. Compound 14d showed high efficacy in an ob/ob mouse model, reducing serum levels of triglycerides, glucose, and insulin. Furthermore, in the evaluation of SGK1 expression in HCCD cells, it reduced gene expression and was able to attenuate relative weight gain. Molecular docking studies confirmed these results [121].

Twenty-eight benzothiazol-1,3,4-oxadiazol-4-thiazolidinones (Tz1–Tz28) were synthesized (Figure 9E), and only seven derivatives (Tz4, Tz10, Tz17, Tz19, Tz21, Tz23, and Tz24) were evaluated in vivo in diabetic rats. After 4 h, hypoglycemic activity was determined using the oral glucose tolerance test (OGTT). Compounds Tz21 (95.35 ± 1.38 mg/dL), Tz17 (95.43 ± 1.33 mg/dL), and Tz10 (101.05 ± 0.70 mg/dL) showed a better hypoglycemic effect than PGZ (100.53 ± 1.35 mg/dL). In molecular docking, all compounds were evaluated as PPAR-γ agonists and showed affinity for the binding ligand. In molecular dynamics, the bond free energies of the evaluated ligands ranged from −47.80 to −59.80 Kcal/mol. Therefore, it was concluded that compounds with electron-donating group substitution on the phenyl ring of 4-thiazolidine exhibit greater antidiabetic activity [122].

Hurtevent et al. designed, synthesized, and evaluated a series of nitrogenous heterocycles containing α-ethoxyphenylpropionic acid derivatives (9–12, 13a, 13b, 15, 16a, 16b, 17a, 17b, 18a, 18b, 19a, 19b, 20a, 20b, 21a, 21b, 22a, 22b, 23, 24a, 24b, 25–30, 31, 32a, 32b, 33–38, 39a, 39b, 40a, 40b, 41a, 41b, 42a, 42b, 43a, 43b, 44a, 44b, 45a, 45b, 46a, 46b) (Figure 9F) as dual PPAR-α/γ agonist ligands for the treatment of T2D and its complications. In vitro studies found that 6-benzoylbenzothiazol-2-one was the most well-tolerated heterocycles tested, in which the incorporation of an *O*-methyl ether oxime and a trifluoroethoxy group, followed by enantiomeric resolution, led to the (S) 44b stereoisomer, which showed the best pharmacological profile. Compound 44b acted as a very potent full compound, a PPAR-γ agonist, and a weak partial agonist for the PPAR-α receptor subtype. It also showed high efficacy in an ob/ob mouse model with significant decreases in serum triglyceride, glucose, and insulin levels, but primarily with limited weight gain [123].

In 2021, Kharbanda et al. evaluated 48 compounds (2a–2x) and (3a–3x) (Figure 9G), and an in vitro PPAR-γ transactivation assay was performed obtaining percentages of 87% for the active compounds of family 2a–2x and between 76% and 86% for family 3a–3x, a good effect compared with rosiglitazone (76.2%). In vivo, they showed a notable reduction in the glucose level in the glucose tolerance test (OGTT), without weight gain or hepatotoxic effects, and the compounds 2n and 3t were found to be the most active. The antidiabetic activity in vitro and in vivo was due to the change in substitutions in the benzothiazolyl and phenyl rings, favoring important high-affinity interactions in molecular docking studies [124].

Alvarado-Salazar et al. examined two families of 2-aminobenzothiazole derivatives, isothioureas (3a–w) and guanidines (4a–z), for the treatment of T2D. In vivo studies demonstrated that compounds 3b and 4y (Figure 9H) were able to reduce blood glucose levels (<200 mg/dL) similarly to PGZ, in addition to having a lipid-lowering effect, without causing liver damage. In an in silico evaluation, these molecules demonstrated a binding affinity for PPAR-γ, first in reverse docking on the DIA-DB online platform and then in targeted molecular docking with a high affinity for PPAR-γ, with molecular recognition at the active site through significant interactions and ΔG values similar to those of reference ligands [125].

Reyes et al. in 2025 evaluated benzothiazole compounds (5a–d, 8a–d, 11a–d, and 12c–d) incorporating amino acid derivatives to enhance the molecules’ (Figure 9I) affinity for both PPAR-γ and aldose reductase (ALR2), the enzyme responsible for the polyol pathway. Determining these properties allowed the selection of the best compounds. Compounds **8c** and **8d** inhibited ALR2 at concentrations below 10 µM. In an in vivo evaluation using a high-fat, STZ-induced diabetic rat model, the compounds successfully controlled fasting blood glucose for 5 weeks. They also reduced insulin resistance, as measured based on the HOMA-IR index and the oral glucose tolerance test, and exhibited an adipogenic effect without altering liver transaminases. In in silico studies, the compounds showed favorable results as PPAR-*γ* agonists. Compound 8d produced sustained antihyperglycemic effects and reduced IR, dyslipidemia, and polydipsia, without inducing hepatotoxicity or exhibiting intrinsic antioxidant or anti-inflammatory activity. These findings suggest that 8d is a promising candidate for further development in diabetes-related therapeutic strategies [117].

### 5.5. Protein Tyrosine Phosphatase 1B (PTP1B)

PTP1B is an intracellular enzyme whose function is the dephosphorylation of tyrosine residues in receptors and adaptor proteins. It plays a key role in the regulation of body weight, glucose homeostasis, and energy expenditure by participating in the negative regulation of insulin and leptin signaling [126,127].

Structurally, PTP1B is a protein of 435 amino acids that is made up of an *N*-terminal catalytic domain, which contains the essential elements for phosphate hydrolysis, and a *C*-terminal region that consists of two zones: a proline-rich region and a hydrophobic segment involved in the anchoring of the enzyme to the membrane of the ER. Within the catalytic domain, the P loop (residues 213–221) contains Cys215, which acts as an essential nucleophile in catalysis. In addition, the WPD loop (Trp179–Asp181) moves between open and closed conformations during substrate binding, with Asp181 being responsible for the protonation of the outgoing group. PTP-1B also has secondary binding sites that amplify ligand interactions and are useful for the design of selective inhibitors, as well as an allosteric site located approximately 20 Å from the catalytic site, formed by residues such as Asn193, Lys197, Phe196, and Phe280, which allows the binding of non-competitive inhibitors [128,129].

The involvement of PTP1B in diabetes and obesity is related to its ability to attenuate insulin signaling via dephosphorylation of the IR and IRS-1, which decreases glucose uptake. It also negatively regulates leptin signaling, favoring obesity. Therefore, PTP1B-deficient mice have an increase in insulin sensitivity and resistance to obesity, which makes this enzyme an attractive therapeutic target for the treatment of T2D and metabolic syndrome. However, the development of selective inhibitors faces significant challenges, given that the catalytic site of PTP1B is highly conserved in other tyrosine phosphatases, which hinders selectivity and increases the risk of side effects. For this reason, allosteric inhibitors or inhibitors that take advantage of secondary sites to improve the specificity of pharmacological compounds are currently being investigated [127].

#### Recent Research on Benzothiazole Derivatives as PTP1B Inhibitors

In 2009, Navarrete et al. determined that compounds from a series of 27 of 2-arylsulfonylaminobenzothiazole derivatives exhibited greater in vitro inhibitory activity against PTP1B, with IC_50_ values at low micromolar concentrations. The intrinsic reversible mixed-type inhibition of these two compounds is attributed to the nitro group, which can engage in hydrogen-bonding interactions with the Arg221 and Ser216 residues within the protein’s catalytic site. They also demonstrated that the intragastric administration of a single 100 mg/kg dose of compounds 4 and 16 (Figure 10A) significantly reduced blood glucose levels in diabetic rats treated with STZ-nicotinamide compared to glibenclamide after 7 h of treatment [126].

Furthermore, in 2012, Navarrete et al. also demonstrated that compounds OX-1, OX-6, and OX-7 (Figure 10B) from a series of eight derivatives, obtained via coupling between 2-amino-6-substituted benzo[d]thiazole and ethyl chlorooxoacetate in a basic medium, exhibit a lower rate of *o*-nitrophenol formation in the colorimetric assay with *p*-nitrophenyl phosphate at 40 mM, suggesting a greater inhibitory effect on PTP1B with IC_50_ values in the low nanomolar range, which is attributed to the fact that the oxamate group establishes hydrogen bond interactions with the amino acids Arg221 and Ser216 of the catalytic site of PTP1B. Between compounds 40c and 40b, the authors observed that 40a exhibited greater hypoglycemic activity (27–37%), while compound 40b maintained an activity profile comparable to that of glibenclamide during the first 3 h after administration of a single 100 mg/kg dose in an acute normoglycemic rat model. Furthermore, both compounds showed an effect similar to the reference drug in the oral glucose tolerance test [130].

Another study conducted by Hidalgo et al. in 2018 demonstrated that compounds 1 and 2 (Figure 10C) exhibit good inhibitory properties against PTP1B at a concentration of 20 μM. The authors observed that **1** reduced enzyme activity by up to 85%, while compound **2** resulted in a 50% decrease. The greater activity of compound 41a is attributed to its higher affinity (ΔG = −8.94 Kcal/mol) for the protein and its maintenance of key interactions with catalytic residues Asp181 and Arg221, as well as additional hydrogen bonds with Arg47 and Asp48. Compound 41a also exhibits good adsorption, distribution, metabolism, excretion, and toxicity (ADMET) properties and has an IC_50_ of 9.6 ± 0.5 μM [131].

In 2019, Maheshwari et al. synthesized ten *N*-(3-(1H-tetrazol-5-yl)phenyl)acetamide derivatives, designed from a lead molecule previously identified by their research group. Compound NM-14 (Figure 10D), which features a chlorine substitution at position 5 of the benzothiazole ring, exhibited the significant inhibition of PTP1B, with a CI_50_ of 1.88 µM, compared to suramin (CI_50_ ≥ 10 µM). In addition to its in vitro activity, 42 demonstrated in vivo antidiabetic efficacy comparable to that observed with standard drugs such as metformin and glimepiride. Therefore, these compounds represent valuable prototypes for the development of novel non-carboxylic PTP1B inhibitors with antidiabetic potential [132].

### 5.6. Glycogen Phosphorylases

Another target is glycogen phosphorylase (GP), the main regulatory enzyme in the liver responsible for the control of blood glucose levels. One of several approaches to influence the action of GP is the use of glucose derivatives as active site inhibitors that might control hyperglycemia in T2D [133,134].

It is vitally important to monitor blood pressure levels, blood cholesterol, and even the amount of adipose tissue, body weight, and the development of early diabetes-related complications, such as kidney disease, retinopathy, and neuropathy. Within all these comorbidities and metabolic complications, different targets are involved, with few considered so far for the treatment of T2D.

#### Recent Research on Benzothiazole Derivatives as Glycogen Phosphorylase Inhibitors

Somsák et al. proposed molecules called azoloperhydropyridines, which can be considered heterocycles fused with an iminosugar, that show moderate inhibition of GP. They reported nojiritetrazol 12 (*Ki* = 53 mM) as the best inhibitor, with a lower number of nitrogen atoms in the five-membered ring, which weakens the bond. They conducted physiological investigations with N-acetyl-β-D-glucopyranosylamine 6, spirothiohydantoin 42, isofagomine 43, and DAB 44 to highlight the potential use of these compounds in the treatment of type 2 diabetes (Figure 11A) [133].

While Chrysina et al. evaluated new analogs of beta-D-glucopyranose, 2-(beta-D-glucopyranosyl)-5-methyl-1, 3, 4-oxadiazole, -benzothiazole, and -benzimidazole (Figure 11B) were assessed for their potency to inhibit GPb activity. The compounds showed competitive inhibition (with respect to substrate Glc-1-P) with *Ki* values of 76 (+/−4.8) (compound **3**) and 8.6 (+/−0.7) (compound 4) μM, respectively. In order to establish the mechanism of this inhibition, crystallographic studies were carried out, and the structures of GPb in complex with the three analogs were determined at high resolution (GPb-methyl-oxadiazole complex, 1.92 Å; GPb-benzothiazole, 2.10 Å; GPb-benzimidazole, 1.93 Å). The benzimidazole bound at the new allosteric inhibitor or indole-binding site, located at the subunit interface, in the region of the central cavity, and also at a novel binding site, located at the protein surface, far removed (approximately 32 Å) from the other binding sites, which are mostly dominated by the nonpolar groups of Phe202, Tyr203, Val221, and Phe252 [134].

### 5.7. 11β-Hydroxysteroid Dehydrogenase Type 1 (11β-HSD1)

Glucocorticoids (GCs) are potent functional antagonists of insulin action and promote hepatic gluconeogenesis. At the tissue level, the access of active GCs to their receptors is regulated by 11β-HSD1.

11β-HSD1 catalyzes the conversion of inactive cortisone into cortisol, a potent functional antagonist of insulin action, and promotes hepatic gluconeogenesis, which could lead to increased blood glucose levels in diabetes [126].

Various evidence has implicated the activity of GCs and 11β-HSD1 in the etiology of central obesity, metabolic syndrome, and the dysregulation of glucose and lipid metabolism [135]. Findings have indicated that it increases gluconeogenesis, compromises insulin secretion, and regulates glucagon release. For these reasons, the inhibition of 11β-HSD1 has been proposed to have potential therapeutic benefits for glucose homeostasis and weight loss, and specific inhibitors of this enzyme have been reported to improve insulin sensitivity among the amino acid residues reported as part of the catalytic site, which are Ser170, Tyr183, Ala172, Ser43, Ile46, Arg66, Ser67, Met93, Asn119, Lys187, Thr222, Ile121, Ala223, Ile218, Leu217, Val227, Ala226, Thr124, Leu126, and Val180 [136].

#### Recent Research on Benzothiazole Derivatives as 11β-HSD1 Inhibitors

In 2006, Su et al. reported the discovery of a series of benzothiazole derivatives (1–17) that were evaluated using a radioimmunoassay (RIA) method in human liver microsomes. Of these, 15 5-arylsulfonamido benzothiazole derivatives exhibited inhibitory activity against 11β-HSD1. In particular, derivatives 1 and 2 (Figure 12A), substituted at position 4 by a chlorine group on the benzothiazole ring, achieved inhibition greater than 80% at 10 μM and showed IC_50_ values in the nanomolar range. The inhibitory capacity of compound 1 was supported by molecular docking analyses, which revealed that the chlorine group is located in the hydrophobic cavity of 11β-HSD1. Furthermore, both the sulfonamide group and the -NH group of the compound 1 establish interaction relationships with the ring and carbonyl of the cofactor, according to their high degree of affinity and inhibitory capacity; therefore, they could have considerable potential in the treatment of metabolic diseases, such as T2D or obesity [137].

An additional study by Moreno-Díaz et al. reported the synthesis of eight *N*-(6-substituted-1,3-benzothiazol-2-yl)benzenesulfonamide derivatives (1–8) (Figure 12B). Most of the compounds showed a hypoglycemic activity profile comparable to that of the standard drug glibenclamide in a non-insulin-dependent diabetes mellitus (NIDDM) model in diabetic rats treated with STZ-nicotinamide. Compounds 3 and 4 showed greater inhibitory capacity against 11β-HSD1 in human embryonic kidney cells (HEK293) at a concentration of 10 μM, due to their ability to form hydrogen bond interactions with the catalytic residues of 11β-HSD1, as demonstrated in an in silico study [135].

On the other hand, in 2020, Cabrera Pérez et al. proposed a series of benzothiazole derivatives substituted with electron-withdrawing and electron-donating groups (C1–C17). They found that compound (*E*)-5-((benzo[d]thiazol-2-ylimino(methylthio)methylamino)-2-hydroxybenzoic acid (C1) (Figure 12C) is an inhibitor of human 11β-HSD1 that was able to decrease the expression of the 11β-HSD1 enzyme better than clobenzorex. In obese Zucker rats (HsdHlr:Zucker-Leprfa/fa), treatment with C1 resulted in decreased plasma cortisol levels, as well as inhibition of the catalytic activity and expression of 11β-HSD1 in mesenteric adipose tissue. C1 has good physicochemical properties and favorable interactions with 11β-HSD1 through hydrogen bonding and hydrophobic interactions at the catalytic site. This suggests that it could be an option to consider due to its results regarding the anti-obesity effect [138].

### 5.8. Aldose Reductase (ALR2)

ALR2 is the limiting enzyme of the polyol pathway, which requires reduced nicotinamide adenine dinucleotide phosphate (NADPH) as a cofactor for reduction reactions. Under normal conditions, its main function is to reduce reactive aldehydes derived from LPO. However, under conditions of hyperglycemia, it displaces up to 30% of glucose, converting it into sorbitol. Sorbitol subsequently causes osmotic stress and, in certain cells, can be transformed into fructose. Therefore, the activation of this pathway gives rise to oxidative stress due to the expenditure of NADPH and the increase in AGEs, which favor the generation of microvascular complications [129,139].

ALR2 is a cytosolic enzyme composed of a single polypeptide chain of 345 amino acid residues. Its active site is located at the *C*-terminal end and is made up of a structural cavity that includes three well-defined subsites. The first of them, called the “anion binding pocket,” is made up of aromatic residues and hydrogen bond donors (Trp20, Tyr48, His110, and Trp111), as well as the cofactor NADPH, essential for its catalytic activity. The second is the hydrophobic pocket formed by Trp20, Phe122, and Trp219 and, finally, the so-called “selectivity pocket” lined by the residues Thr113, Phe122, Trp111, Cys298, Leu300, and Cys303 [129].

The inhibition of this enzyme has begun to be studied treat diabetic complications; however, there are certain complications since the main structural characteristic required for this activity is the presence of carboxylic acids, which usually have low cellular permeability and limited bioavailability, hindering their absorption and, therefore, their therapeutic efficacy in vivo.

#### Recent Research on Benzothiazole Derivatives as ALR2 Inhibitors

Aotsuka et al. synthesized a series of 24 benzothiazol-2-carboxylic acids with various spacers between the carboxyl group and the benzothiazole ring (4a–d, 5a–g, 6a–i and their congeners 14, 20, 22a and GP-1447) (Figure 13A) and evaluated their biological activity in inhibiting ARL2 in porcine crystallin using D-glyceraldehyde as a substrate, as well as the percentage of inhibition of sorbitol accumulation in the tissues of STZ-induced diabetic rats. The studies showed that the most potent compounds belonged to the 6a-i series, with 6a and 6i being particularly prominent. Compound 6a exhibits greater inhibition of ALR2 (IC_50_ = 6.6 nM) compared to zenarestat (IC_50_ = 12 nM) and SG-210 (IC_50_ = 12 nM). However, compound 6i demonstrates the best in vivo activity, with ED_50_ = 0.12 mg/kg in the sciatic nerve and 0.71 mg/kg in the lens of the eye, compared to SG-210 (EC_50_ = 1.8 mg/kg and EC_50_ > 10 mg/kg, respectively). Furthermore, compound 6b (GP1447**)** was selected as a candidate for further pharmacological evaluations due to its good oral absorption, low toxicity, and high ALR2 inhibitory potency (IC_50_ = 9.6 nM) and high selectivity influenced by the inhibition (EC_50_ = 1.8 mg/kg) of sorbitol in the sciatic nerve and lens (EC_50_ > 10 mg/kg) of diabetic rats [140].

Rakowitz et al. synthesized a 24-compound series targeting ALR2 inhibition. They evaluated how different structural modifications impact their biological activity. Within this series, benzothiazole oxime derivatives (2a/b) were synthesized as *E*-bioisosteres of carboxylic acids and evaluated as ALR2 inhibitors using DL-glyceraldehyde and NADPH. In comparison, the oximes showed better activity, particularly 2a (IC_50_ = 44.9 µM) compared to 2b (38% at 50 µM), confirming that bioisosteric substitution of the acid group with an oxime can enhance inhibition (Figure 13B1,B2) in comparison to sorbinyl (IC_50_ = 1.2 µM). The benzothiazole core is important for enzymatic activity; the results demonstrate that bioisosterism and substituent optimization allow for a significant improvement in inhibitory activity [141].

Kumar et al. highlight that, although zopolrestat is more potent (IC_50_ = 3.1 nM), its low selectivity against ALR2 limits its usefulness. In contrast, they indicate that fidarestat maintains high potency (IC_50_ = 9 nM) along with high selectivity (133-fold), making it safer and more therapeutically relevant (Figure 13C). Furthermore, they indicate that the benzothiazole-derivative compound 12 has high selectivity and potency (IC_50_ = 0.04 ± 0.02 μM) against ALR2. The high selectivity is attributed to the benzothiazole ring fitting into the specificity pocket [142].

Kousaxidis et al. conducted a literature review that included evaluating IC_50_ values, selectivity against ALR2, and pharmacokinetic properties. They identified a wide variety of compounds with ALR2-inhibitory activity. In the series of compounds with a benzothiazole moiety, they identified compounds 6a and 6b, which incorporate a pyrrolylacetic acid group, as having IC_50_ = 49 and 45 nM, respectively. They also identified that Van Zandt et al. developed 3-[(benzothiazol-2-yl)methyl]indole-N-alkanoic acid derivatives, where compounds with a trifluorobenzothiazole group (6a-i) exhibited even greater activity (IC_50_ = 5–8 nM), with the methoxy group insertion at position 6 of the indole fragment 6g (IC_50_ = 5 nM) being the most potent in comparison to Lidorestat. Crystallographic studies demonstrated that the carboxylate forms several hydrogen bonds at the enzyme’s catalytic site. Furthermore, the benzothiazole moiety induces conformational changes in the hydrophobic cavity by opening the selectivity pocket, which explains its high selectivity (Figure 13D) [129].

Server et al. synthesized a series of 18 new compounds derived from 2-[(4-amino-5-aryl-4H-1,2,4-triazol-3-yl)thio]-N-(thiazole/benzothiazol-2-yl)acetamides and evaluated their activity as ALR2 inhibitors by measuring the decrease in NADPH absorbance in the presence of DL-glyceraldehyde. They also determined their cytotoxicity using the MTT method and their affinity for the enzyme via molecular docking. The results indicated that compound 17 (IC_50_ = 0.311 μM and Ki = 0.08 ± 0.02 μM) exhibited excellent ARL2-inhibitory activity compared to quercetin (IC_50_ = 3.86 μM and Ki = 5.66 ± 0.66 μM) (Figure 13E). Furthermore, they demonstrated that this effect can be attributed to their interactions with Trp219, Phe122, and Trp111 via hydrogen bonding, π-π interactions, and hydrophobic interactions, as determined via molecular docking studies. Furthermore, they demonstrated that the compounds do not cause cytotoxicity in L929 cells at the IC_50_ values determined for their ARL2-inhibitory activity, thus demonstrating their safety as AR inhibitors [86].

Reyes-Vallejo et al. studied a series of 14 2-aminobenzothiazole derivatives (Figure 13F) as potential inhibitors of ALR2. All compounds showed a good pharmacological profile, with compounds **8d** and **8c** standing out due to their higher affinity (ΔG = −8.39 Kcal/mol and −8.14 Kcal/mol, respectively) for the enzyme via molecular docking. Experimentally, compounds 8c (<50%) and 8d (<80%) showed a better percentage inhibitory effect on ALR2 (<80%) than epalrestat in a concentration range of 10,000 to 5 nM. This is because the benzothiazole core interacts with the cofactor and residues of the anion-binding pocket (His110 and Trp111) through π–π, π–alkyl, and π–sulfur interactions [117].

### 5.9. Other Potential Targets for T2D Treatment

#### Inhibitors of Apoptosis Signal Regulating Kinase 1

Meltzer-Mats et al. designed and synthesized 34 benzothiazole derivatives as targeting the AMPK activator PT-1, with the aim of developing new agents with antihyperglycemic activity. Based on biological assays, compound 34 stood out as the most active, significantly increasing glucose uptake in L6 myotubes (up to 2.5–fold) and promoting GLUT4 translocation through direct AMPK activation, independent of the insulin pathway (PI3K/AKT). Pharmacophoric analysis revealed that the ethoxybenzothiazole moiety is key to this activity, possessing hydrophobic, aromatic, and hydrogen-bond-accepting characteristics that favor its interaction with the enzyme. Furthermore, the compound induced the phosphorylation of downstream proteins such as AS-160 and ACC, confirming the functional activation of the AMPK pathway. These in vitro results correlated with in vivo studies, where compound 34 (Figure 14A) reduced glucose levels in KKAy diabetic mice, positioning it as a promising antidiabetic candidate and highlighting benzothiazole as a key scaffold in the rational design of AMPK activators [89].

Starosyla et al. performed the virtual screening of 270,000 compounds using semi-flexible molecular docking with the DOCK software, focusing on the ATP-binding site of Apoptosis Signal Regulator Kinase 1 (ASK1), followed by validation using in vitro assays. Of the 186 selected compounds, a novel class of inhibitors based on benzothiazol-2-yl-3-hydroxy-5-phenyl-1,5-dihydropyrrole-2-one was identified. QSAR analysis revealed that the benzothiazole core is essential for inhibitory activity due to its hydrophobic interactions and a key π-sulfur interaction with residues in the active site (such as Val757). Furthermore, activity is strongly dependent on the R1 substituent, where increased hydrophobicity enhances potency. Among all the derivatives, compounds 27 and 34 stood out as the most potent, with IC_50_ values of 2.5 μM and 0.52 μM, respectively, positioning themselves as the best inhibitors in the series. Overall, this work demonstrates that virtual screening combined with experimental validation allows for the identification of selective ASK1 inhibitors that may be useful for the treatment of T2D, highlighting benzothiazole as a key scaffold in rational drug design (Figure 14B) [143].

Cabrera Pérez et al. recently demonstrated that compound C1 (Figure 14C) significantly reduced body weight and growth rate in obese Zucker rats after 28 days of treatment, an effect associated with activation of the β3-adrenergic receptor (β3-ADR) and stimulation of uncoupling protein 1 (UCP-1), promoting thermogenesis. This behavior is supported by molecular docking studies, where C1 showed key interactions, primarily through hydrogen bonds between its hydroxyl and secondary amine groups with residues such as Asp117, Val121, and Thr122 at the receptor’s active site, helping to elucidate its binding mode and agonist activity. Concomitantly, this activation was associated with a significant decrease in free fatty acids and TNF-α, as well as an increase in glutathione levels, suggesting an improvement in redox status. A trend toward reduced LPO and IL-6 levels was also observed. Additionally, an increase in blood glucose levels was reported, accompanied by a decrease in insulin, without inducing a diabetic state. Taken together, these results indicate that the modulation of β3-ADR by C1 impacts energy metabolism, reduces oxidative stress and inflammation, and may exert a hepatoprotective effect, although with possible implications for glucose homeostasis [144].

### 5.10. Integrated Analysis of the Structure–Activity Relationship of Benzothiazole Derivatives

The benzothiazole derivatives analyzed here indicate that antidiabetic activity cannot be attributed solely to the benzothiazole core, but rather to the combined influence of the substitution pattern, electronic properties, steric requirements, molecular hybridization, and the specific structural requirements of each molecular target. Although substitution at the C-2 position has traditionally been recognized as a key determinant of the biological versatility of benzothiazoles, modifications at other positions of the fused ring particularly at positions 5 and 6, as well as changes in the attached aromatic and heterocyclic groups, can significantly influence potency and target selectivity [76,85,102,105]. Consequently, the SAR trends observed in these derivatives should be interpreted in the context of the specific target and molecular scaffold, rather than as universal rules for benzothiazole-based antidiabetic agents.

Regarding the inhibition of α-glucosidase and *α*-amylase, both the nature and the position of the substituents appear to be critical determinants of activity. In the benzothiazole–triazole series described by Gong et al., compound 6s, featuring a chlorine atom at the 5-position of the benzothiazole ring and a tert-butyl group at the *para* position of the phenyl ring, exhibited the highest *α*-glucosidase inhibitory activity in the series (IC_50_ = 20.7 μM). Halogen substitution contributed favorably to inhibitory activity within this particular structural series [12]. In contrast, benzothiazole–rhodanine derivatives displayed a different structure–activity relationship (SAR) pattern: electron-donating groups at specific positions on the phenolic ring were associated with improved *α*-amylase and *α*-glucosidase inhibition, whereas electron-withdrawing substituents, such as Cl, Br, and NO_2_, were generally associated with lower activity [102]. Likewise, thiazolidinone-based benzothiazole derivatives demonstrated that the presence and position of fluorine and hydroxyl substituents could enhance the inhibition of both enzymes, with compounds 4–6 exhibiting greater potency than acarbose [76].

The importance of substitution topology is supported by sulfur-substituted (*S*-position) benzothiazole-thiazole bis-Schiff base derivatives, in which the position, size, steric contribution, and electronic characteristics of the phenyl ring substituents notably influenced inhibitory potency. In this series, *para* substitution was generally more favorable than *ortho* substitution, and the presence of a *para*-trifluoromethyl group was associated with particularly potent inhibitory activity [105]. However, this behavior should not be generalized to all benzothiazole derivatives. For example, in polyphenolic benzothiazoles evaluated as α-glucosidase inhibitors, hydroxyl groups participating in interactions with enzyme residues were important for activity, whereas fluorine substitution did not necessarily enhance inhibition [106]. Taken together, these findings indicate that electronic effects alone are insufficient to predict antidiabetic potency; steric accessibility, substitution position, and the three-dimensional orientation of the substituent within the corresponding binding site are also critical factors [76,102,105,106].

Comparable behavior dependent on the structural scaffold is observed among PPAR-targeting benzothiazoles. In benzothiazole-1,3,4-oxadiazole-4-thiazolidinone hybrids, substitution with electron-donating groups on the phenyl portion of the thiazolidinones moiety was associated with enhanced antidiabetic activity; notably, compounds Tz21, Tz17, and Tz10 demonstrated the most favorable in vivo hypoglycemic effects among the evaluated derivatives [122]. Structural optimization of α-alkoxyphenylpropionic acid derivatives containing a 6-benzoylbenzothiazol-2-one scaffold further demonstrated the importance of both substituent identity and stereochemistry. The incorporation of an O-methyloxime ether and a trifluoroethoxy group, followed by enantiomeric resolution, yielded the (*S*)-44b stereoisomer; this compound exhibited the most favorable pharmacological profile acting as a potent full PPAR-γ agonist and a weaker partial PPAR-α agonist along with beneficial in vivo metabolic effects [124]. Other PPAR-γ-targeting series containing benzothiazole similarly indicate that modifications to the substitution patterns of the benzothiazolyl and phenyl rings can substantially alter receptor interactions and biological activity [123].

In the case of PTP1B inhibition, halogenation can also contribute positively when incorporated within an appropriate structural context. Maheshwari et al. identified NM-14, a tetrazole-acetamide derivative featuring a chlorine atom at the 5-position of the benzothiazole ring as the most potent compound in their series, exhibiting an IC_50_ of 1.88 μM against PTP1B and in vivo antidiabetic activity comparable to that of metformin and glimepiride [132]. However, the divergent effects of halogen substitution observed in α-glucosidase and α-amylase inhibitor series reinforce the conclusion that chlorine, fluorine, bromine, or other electron-withdrawing substituents cannot be considered universally favorable modifications. Their contribution depends on the overall molecular architecture and on whether the substituent enhances hydrophobic contacts, steric complementarity, the molecular orientation, or other specific interactions with the target [12,76,102,105,106,132].

The series of ALR2 inhibitors provides a further example of the importance of spatial organization and the identity of functional groups. Aotsuka et al. demonstrated that modifying the spacer located between the benzothiazole core and the carboxylic acid group substantially altered inhibitory potency against ALR2 and oral activity. Among the benzothiazol-2-yl-carboxylic acids evaluated, derivatives containing a phenylacetic acid unit proved particularly potent, with compound GP-1447 standing out as a highly potent, orally active inhibitor [140]. In a related study, Rakowitz et al. investigated the bioisosteric replacement of the acidic carboxyl function and modification of the lipophilic region. Substitution with an oxime group altered inhibitory activity against ALR2, demonstrating that both the acidic pharmacophoric region and the lipophilic substituent contribute to enzyme recognition [141]. These results illustrate that the linker length, spatial arrangement, and bioisosteric substitution can be just as important as a simple aromatic substitution in determining the activity of benzothiazole derivatives against diabetes-related targets [140,141].

Taken together, the available SAR evidence suggests several recurring, though not universal, principles. First, the C-2 position represents a key site for functionalization and pharmacophore attachment, while substitutions at the 5 and 6 positions can further modulate potency and selectivity [12,76,85,132]. Second, the position of substituents on attached aromatic rings can be critical; *para* substitution is advantageous in some series, whereas *ortho* substitution may introduce unfavorable steric constraints [105]. Third, halogenation can either enhance or reduce activity, depending on the molecular structure and the target [12,76,102,105,106,132]. Fourth, electron-donating groups appear favorable in several series targeting α-amylase or α-glucosidase and PPAR, although this trend cannot be generalized to all benzothiazoles [102,122]. Finally, molecular hybridization with thiazolidinedione, rhodanine, triazole, oxadiazole, thiazole, and related pharmacophores provides additional interaction sites and can substantially modify biological and physicochemical properties [12,76,102,105,122]. Overall, these observations support the benzothiazole core as a versatile structural platform rather than a standalone antidiabetic pharmacophore; its pharmacological behavior ultimately depends on the complete molecular architecture and the structural requirements of the biological target.

To complement the target-focused analysis and facilitate comparisons among the most representative benzothiazole derivatives, Table 1 summarizes the compounds and structural series selected based on their primary molecular target, the level and type of experimental evidence, the experimental model, and the most relevant pharmacological findings. Given that studies differ considerably regarding assay design, experimental conditions, parameters evaluated, doses, concentrations, and reported units, the table aims to provide a qualitative and contextual overview rather than a direct quantitative ranking of pharmacological potency across the various studies.

## 6. Conclusions

The evidence analyzed in this work supports the benzothiazole core as a versatile scaffold in medicinal chemistry for the design of compounds with potential relevance to T2D and its associated metabolic disorders. Benzothiazole-containing derivatives have been investigated against multiple molecular targets involved in glucose homeostasis and metabolic regulation, including α-amylase, α-glucosidase, DPP-4, PPAR-γ, PTP1B, glycogen phosphorylase, 11β-HSD1, ALR2, AMPK-related pathways, and other relevant targets. This diversity is particularly relevant given the multifactorial nature of T2D and the need to explore compounds capable of modulating complementary metabolic mechanisms.

The structure–activity relationships analyzed throughout this review indicate that the biological activity of these compounds cannot be attributed solely to the benzothiazole core. Rather, activity depends on the overall molecular architecture and the specific structural requirements of the target. Functionalization at C-2, substitution at positions 5 and 6, the position and electronic nature of substituents, halogenation, the characteristics of linker groups, and molecular hybridization with other pharmacophores can substantially influence potency, selectivity, and physicochemical properties. Consequently, benzothiazole should be considered an adaptable structural platform rather than a universal antidiabetic pharmacophore.

Several benzothiazole derivatives have demonstrated activities comparable to or, in some experimental settings, superior to those of the reference compounds used in the respective studies, thereby supporting the pharmacological relevance of this scaffold. However, these findings must be interpreted with caution, as the available evidence stems predominantly from silico, in vitro, ex vivo, and preclinical in vivo studies. Furthermore, differences in experimental models, testing conditions, dosages, endpoints, and reported units limit direct comparisons between studies, and the clinical efficacy of benzothiazole derivatives for T2D has not yet been established.

Therefore, future research should focus on the most promising derivatives through integrated target validation, selectivity assessment, pharmacokinetic and toxicological characterization, and well-designed in vivo studies evaluating efficacy and safety following repeated exposure. Such studies will be necessary to determine whether the favorable preclinical properties identified in selected benzothiazole derivatives can translate into viable candidates for the further development of antidiabetic drugs.

## Figures and Tables

**Figure 1:**
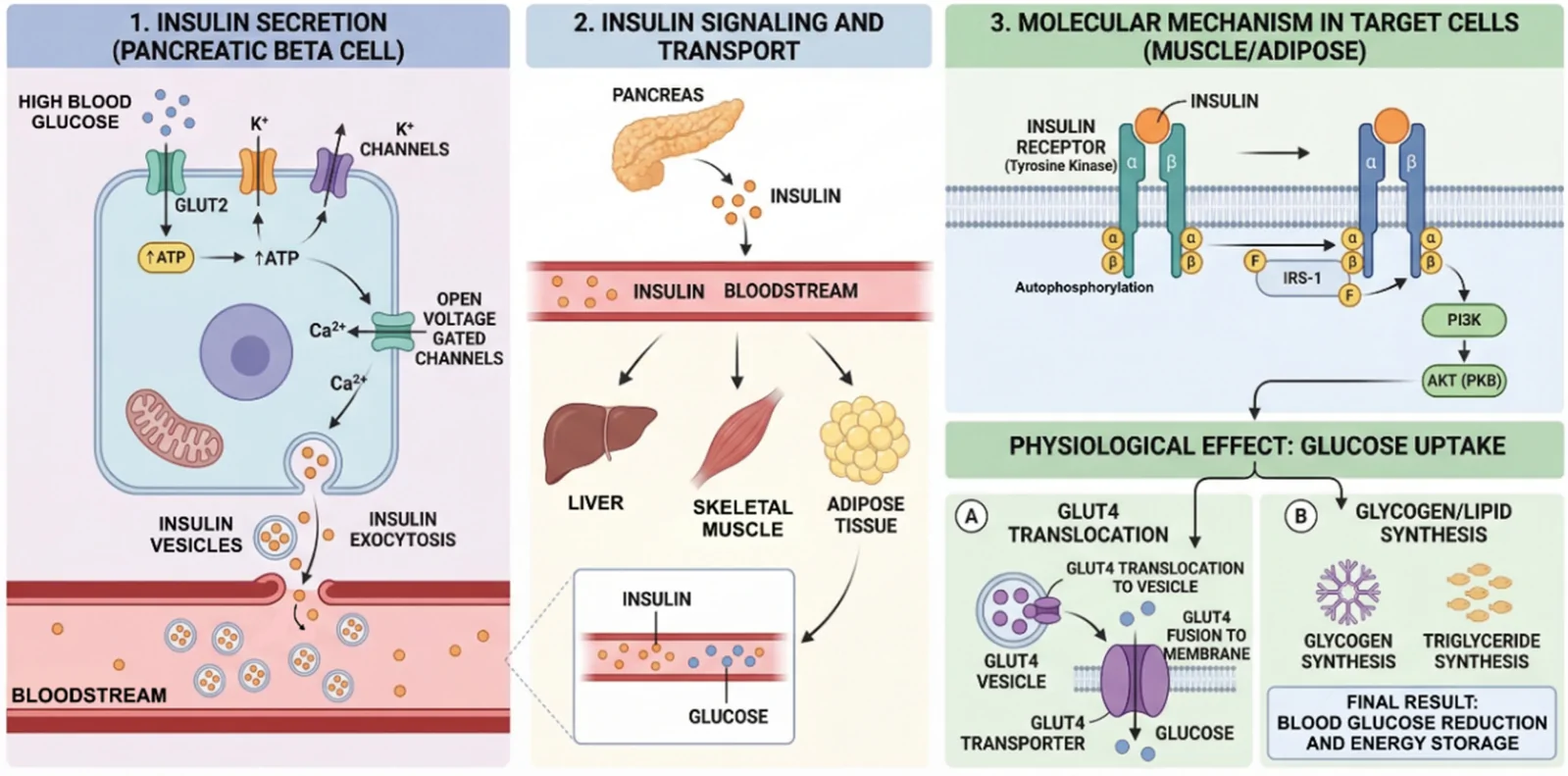
Molecular mechanism of insulin: secretion, transport, and cellular action. ATP: adenosine triphosphate, Ca^2+^: calcium ion, K^+^: potassium ion. (Image generated using Gemini (Google) and reviewed/adapted by the authors).

**Figure 2:**
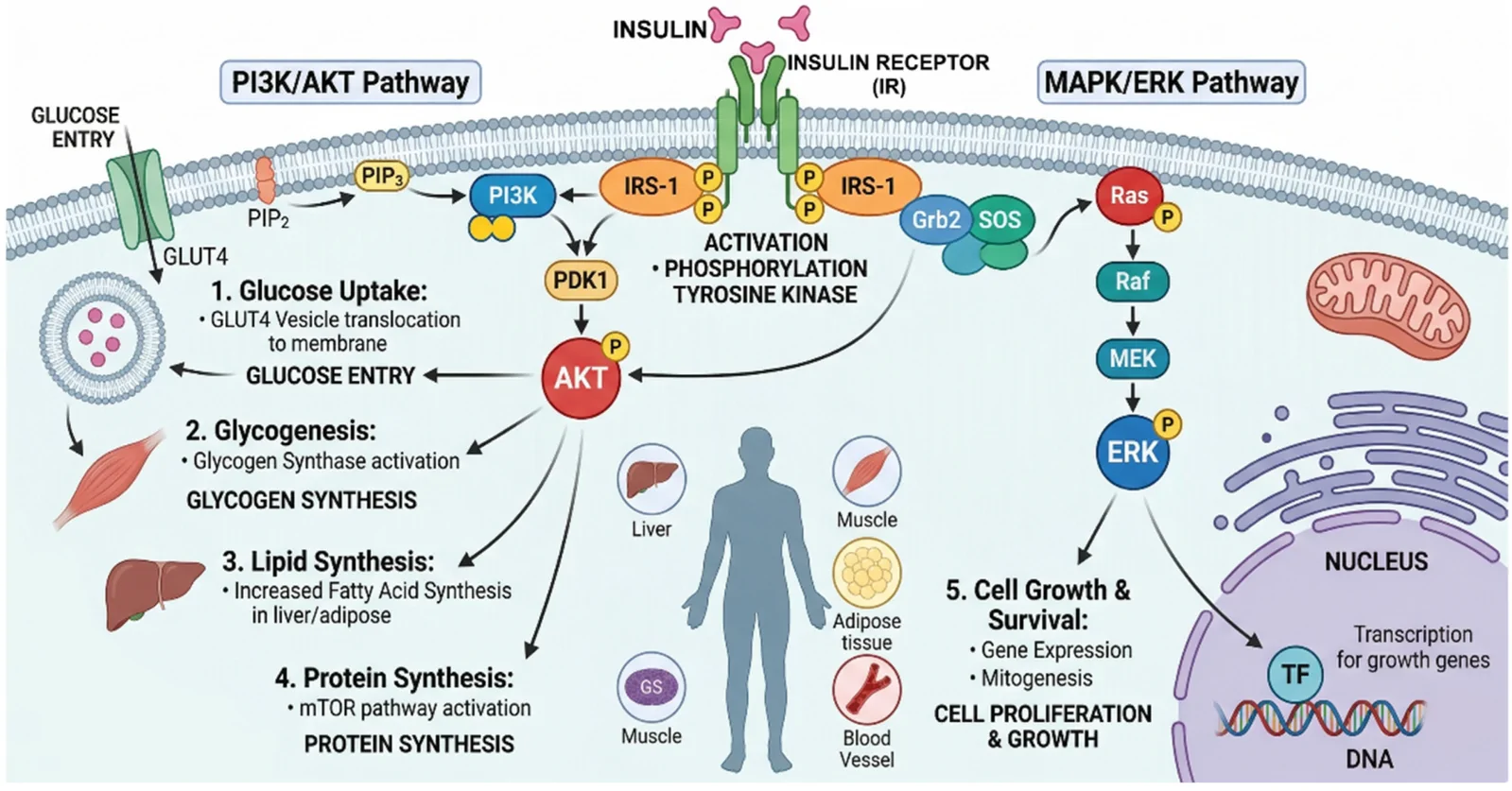
Schematic representation of the insulin signaling pathway, including PI3K/AKT and MAPK/ERK cascades and their physiological effects. (Image generated using Gemini (Google) and reviewed/adapted by the authors).

**Figure 3:**
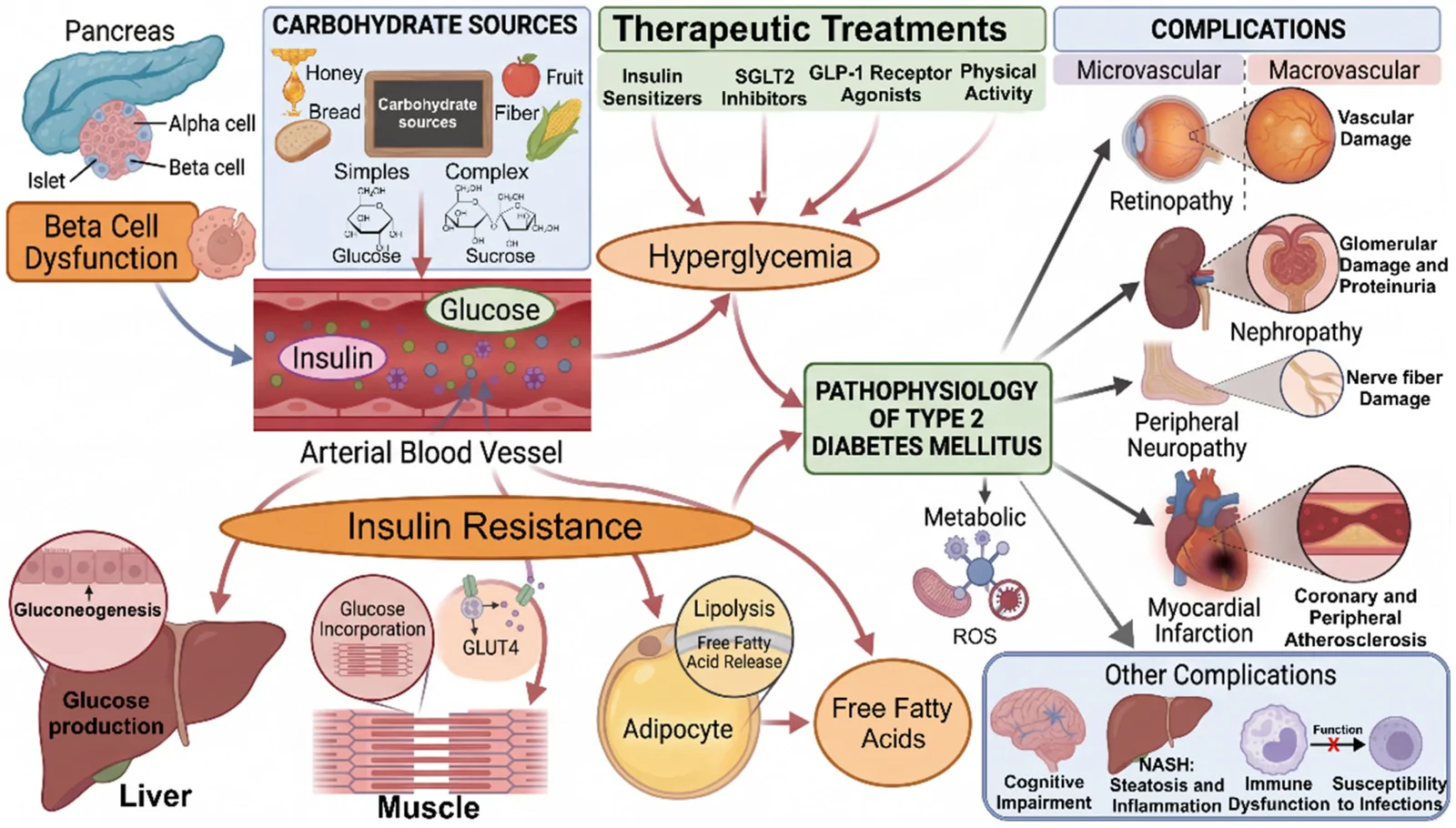
Mechanisms involved in the pathophysiology of T2D and its comorbidities associated with chronic hyperglycemia. (Image generated using Gemini (Google) and reviewed/adapted by the authors).

**Figure 4:**
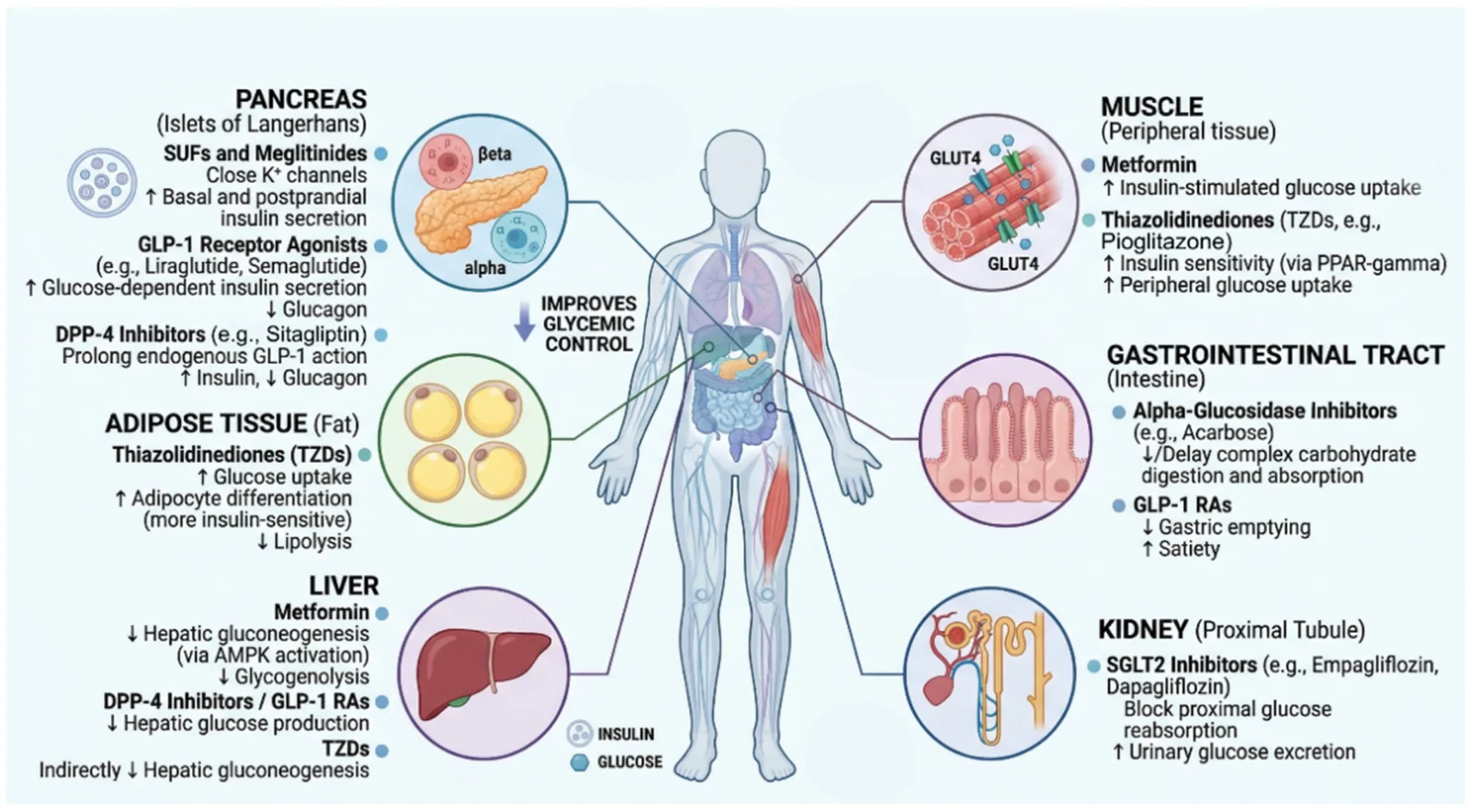
Pharmacological targets and mechanisms of antidiabetic drugs in peripheral tissues and organs. (Image generated using Gemini (Google) and reviewed/adapted by the authors).

**Figure 5:**
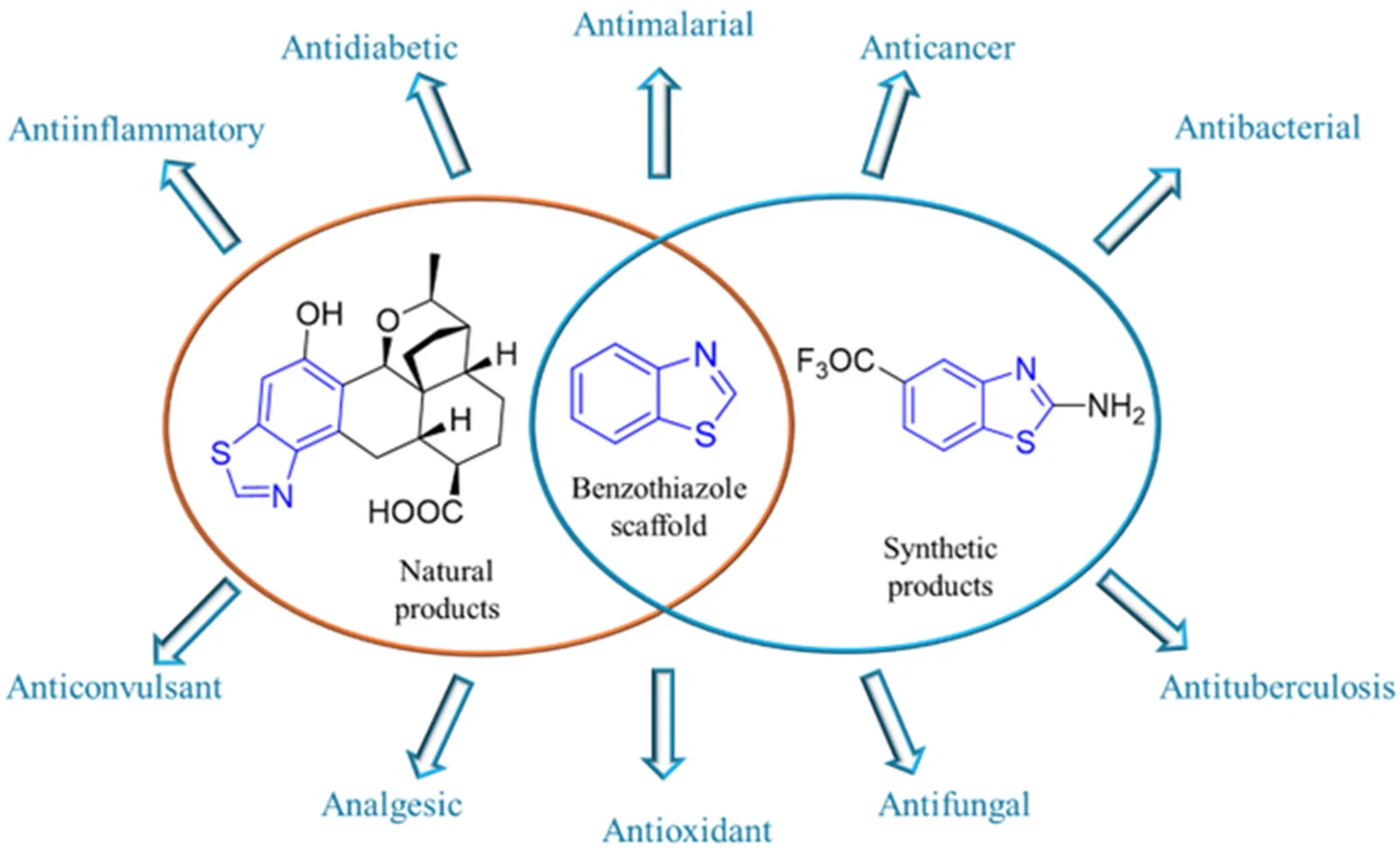
Importance of benzothiazoles and their different biological activities.

**Figure 6:**
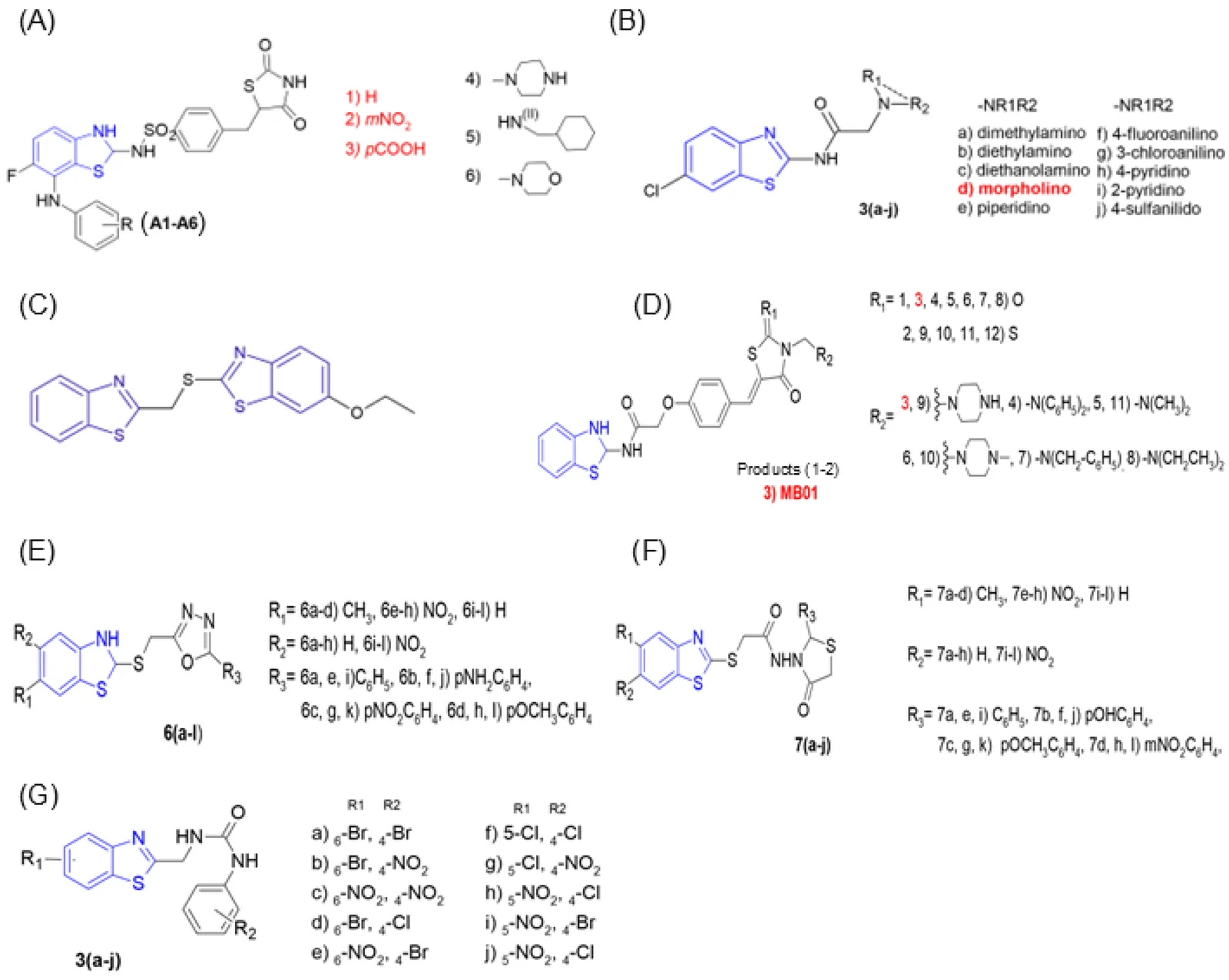
Compounds derived from 2-amino [5’(4-sulphonylbenzylidine)-2,4-thiazolidinenone]-7-chloro-6-flurobenzothiazole (**A**) [87]. Compounds derivates of N-(6-chlorobenzothiazol-2-yl)-2-(substituted amino) acetamides (**B**) [88]. Molecule 2-(benzo[d]thiazol-2-ylmethylthia)-6-ethoxybenzo[d]thiazo (**C**) [89]. Molecules mixing 2-aminobenzothiazole and thiazolidinedione (**D**) [90]. Chemical structures of benzothiazoles bound to oxadiazoles (**E**) [91]. Compounds containing benzothiazoles (**F**) [92]. Compounds derivate of urea bound to benzothiazole to which a series of electron acceptors was incorporated as an antidiabetic (**G**) [93].

**Figure 7:**
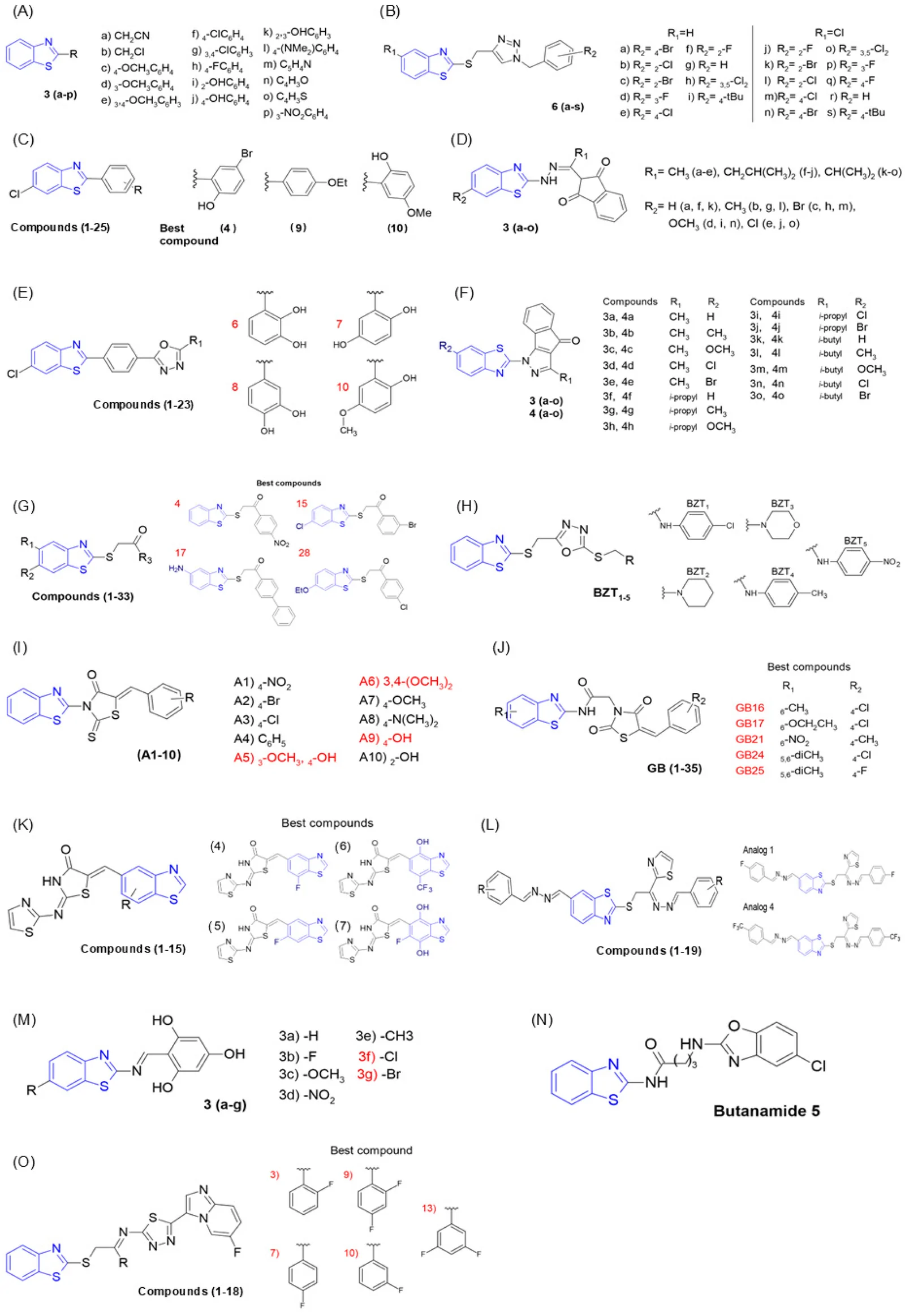
Compound derivatives from benzothiazole (**A**) [94]. Compounds with a triazole-based structure fused to a benzothiazole (**B**) [12]. Small molecules derived from 5-chloro-2-aryl benzo[d]thiazole (**C**) [95]. Compounds derived of benzothiazolyl hydrazones (**D**) [98]. R-(5-(4-(6-chlorobenzo[d]thiazol-2-yl) phenyl)-1,3,4-oxadiazol-2-yl)benzene-1,3-diol (**E**) [99] and benzothiazoles with Br or Cl substituents in position 5 with potential inhibition of α-glucosidase (**F**) [100]. Compounds derived from 2-mercaptobenzotiazole (**G**) [101]. Benzothiazole derivatives bridged with indenedione through a methylenehydrazone linker (**H**) [102]. Evaluated benzothiazole-rhodanine derivatives and pharmacophore map descript (**I**) [103]. Novel N-substituted 5-benzylidene-2,4-thiazolidinedione derivatives, based on the antidiabetic effect of TZDs through a peroxisome proliferator-activated receptor gamma (PPAR-γ) (**J**) [104]. Molecules obtained via the functionalization of the other carbon atoms modify one of the carbonyl groups (**K**) [76]. S-substituted benzothiazole derivatives incorporating thiazole rings and bis-Schiff bases (**L**) [105]. Compounds obtained by reacting the 6-substituted-2-aminobenzothiazole compounds with 2,4,6-trihydroxybenzaldehyde (**M**) [106]. Compound *N*-(benzothiazole-2-yl)-4-((5-chlorobenzoxazole-2-yl) aminobutanamide (**N**) [107]. A series of derivatives integrating a fluor-substituted imidazopyridine structure with a thiadiazole moiety retaining the S-substituted benzothiazole structure (**O**) [108].

**Figure 8:**
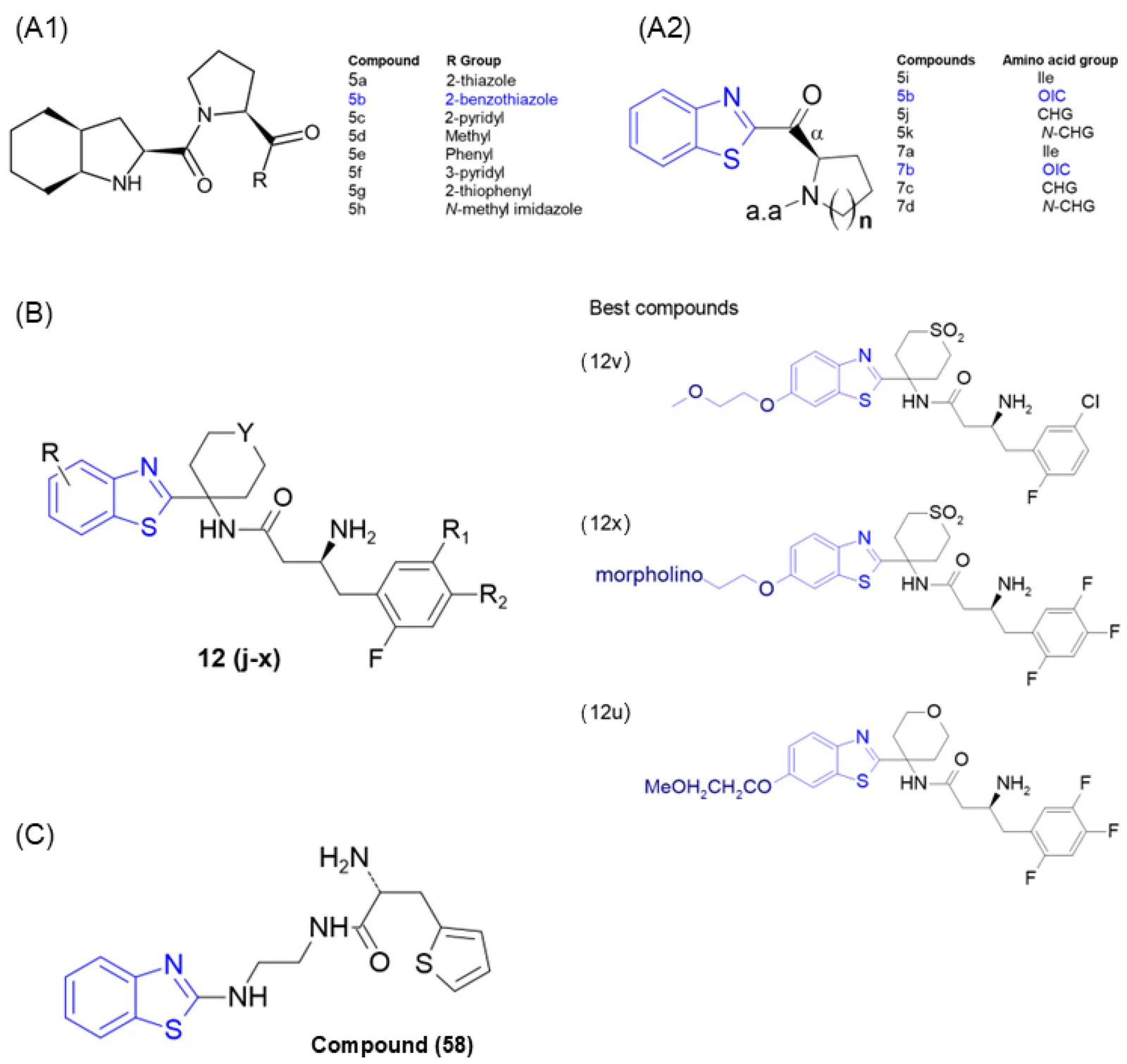
Compounds were studied: ketopyrrolidines and ketoazetidines fused with chemical structures of benzothiazoles (**A1**,**A2**) [112]. 3-Amino-N-(4-aryl-1,1-dioxothian-4-yl)butanamides and 3-amino-N-(4-aryltetrahydropyran-4-yl)butanamides as DPP-4 inhibitors, 15 of which contained a benzothiazole moiety (**B**) [113]. Molecule 58, which contains benzothiazole and benzoxazole, was the best (**C**) [114].

**Figure 9:**
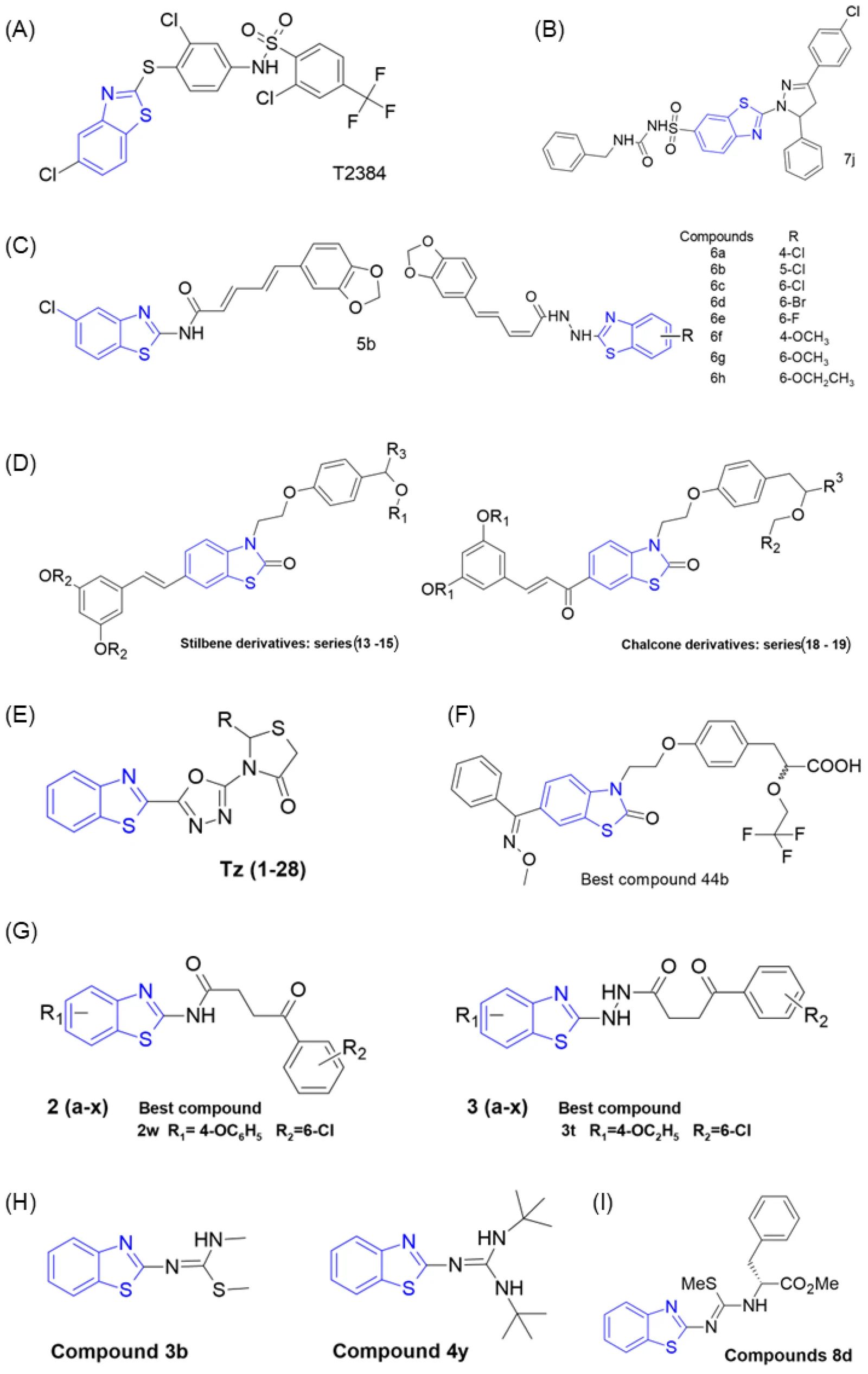
Chemical structures of T2384 (2-chloroN-(3-chloro-4-(5-chlorobenzo[d]thiazol-2-ylthio)phenyl)-4-(trifluoromethyl)benzenesulfonamide) (**A**) [11]. Molecule of sulfonylurea/sulfonylthiourea compounds with benzothiazole moiety (**B**) [119]. Benzothiazole derivatives for PPAR-*γ* agonism (**C**) [120]. Benzothiazol-2-one containing a-ethoxyphenylpropionic acid derivatives incorporating resveratrol or butein scaffolds (**D**) were designed as fused full PPAR-γ agonist ligands and SIRT1-activating compounds for T2D [121]. Benzothiazol-1,3,4-oxadiazol-4-thiazolidinones (**E**) [122]. 6-Benzoyl-benzothiazole-2-one (**F**) [123]. Best compounds that contain benzothiazol (**G**) [124]. Compounds of guanidinobenzothiazoles and benzothiazol isothioureas compounds (**H**) were evaluated in a rat model with STZ-induced T2D, which have an anti-hyperglycemic effect, similar to PGZ [125]. Benzothiazozolisothiourese compounds (**I**) were evaluated, but on this occasion, amino acid derivatives were incorporated in order to improve the affinity of the molecules, both based on PPAR-γ and on aldose reductase, the enzyme responsible for the polyol pathway [117].

**Figure 10:**
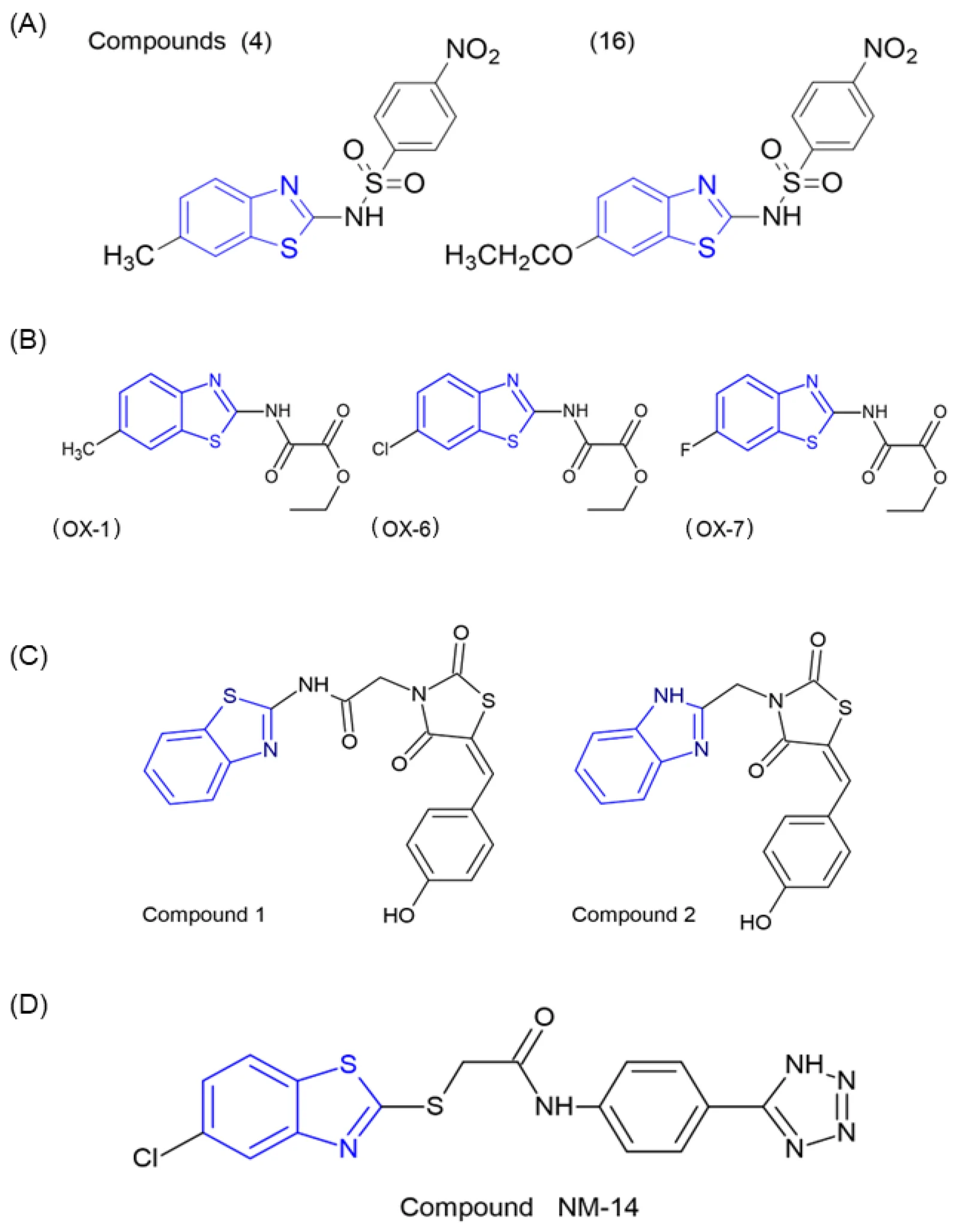
Chemical structure of 2-arylsulfonylaminobenzothiazole derivatives (**A**) [126]. Chemical structure of ethyl 2-(6-substituted benzo[d] thiazol-2-ylamino)-2-oxoacetates derivates (**B**) [130]. *N*-(1,3-benzo[d]thiazol-2-yl)-2-[(5Z)-5-(4-hydroxybenzylidene)-2,4-dioxo-1,3-thiazolidin-3-yl] acetamide derivatives (**C**) [131]. Chemical structure of tetrazole acetamide derivative (**D**) [132].

**Figure 11:**
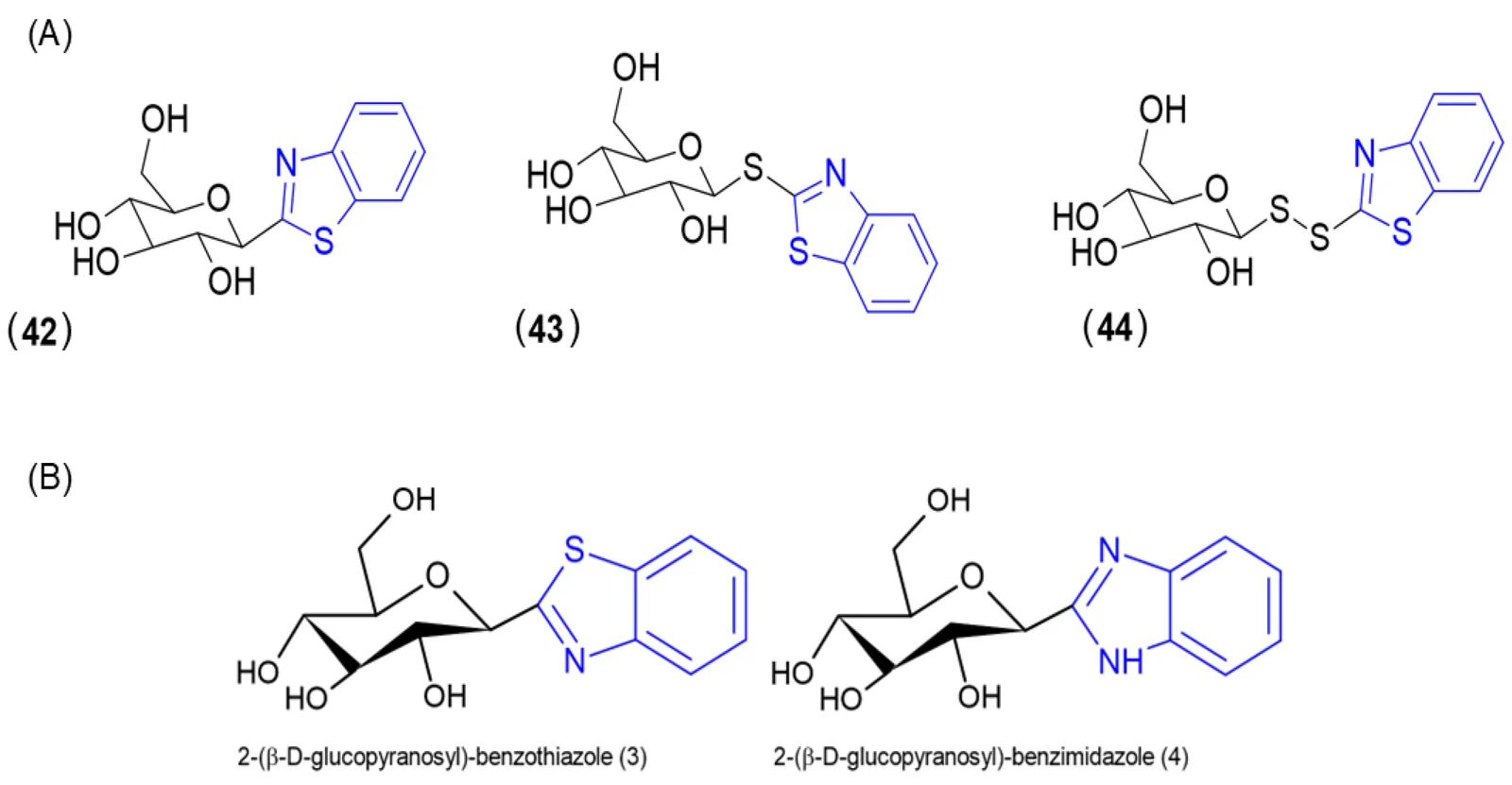
C-(*β*-D-glucopyranosyl) (43a), *β*-D-glucopyranosylsulfanyl (43b), and -disulfanyl heterocycles (**A**) [133]. Analogs of beta-D-glucopyranose, 2-(beta-D-glucopyranosyl)-5-methyl-1, 3, 4-oxadiazole, -benzothiazole, and -benzimidazole (**B**) [134].

**Figure 12:**
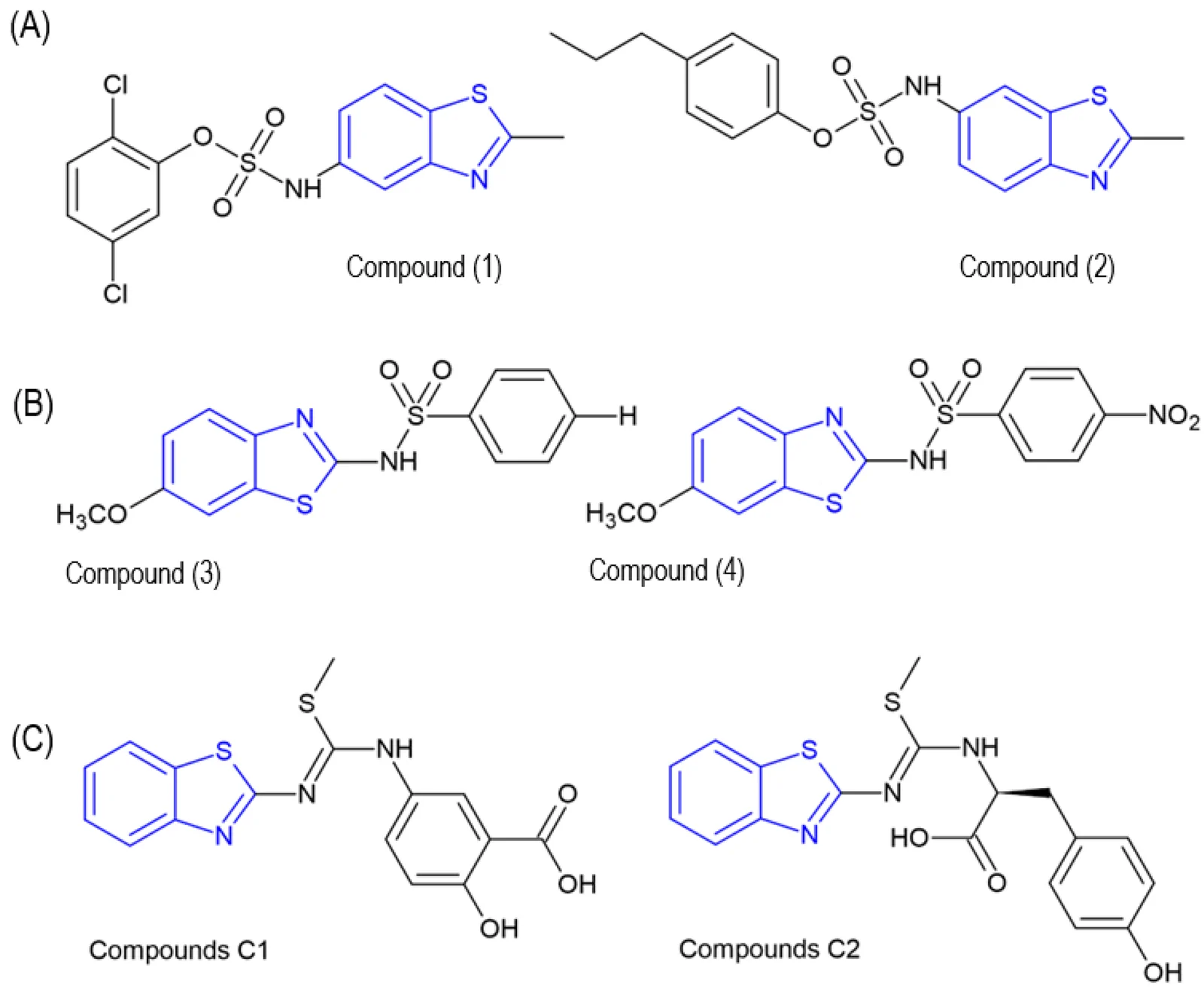
Compounds of 5-arylsulfonamido benzothiazole derivatives (**A**) [137]. Chemical structures of 11β-HSD1 inhibitors derived from *N*-(6-substituted-1,3-benzothiazol-2-yl) benzenesulfonamides (**B**) [135]. Chemical structure of benzothiazole derivates (**C**) [138].

**Figure 13:**
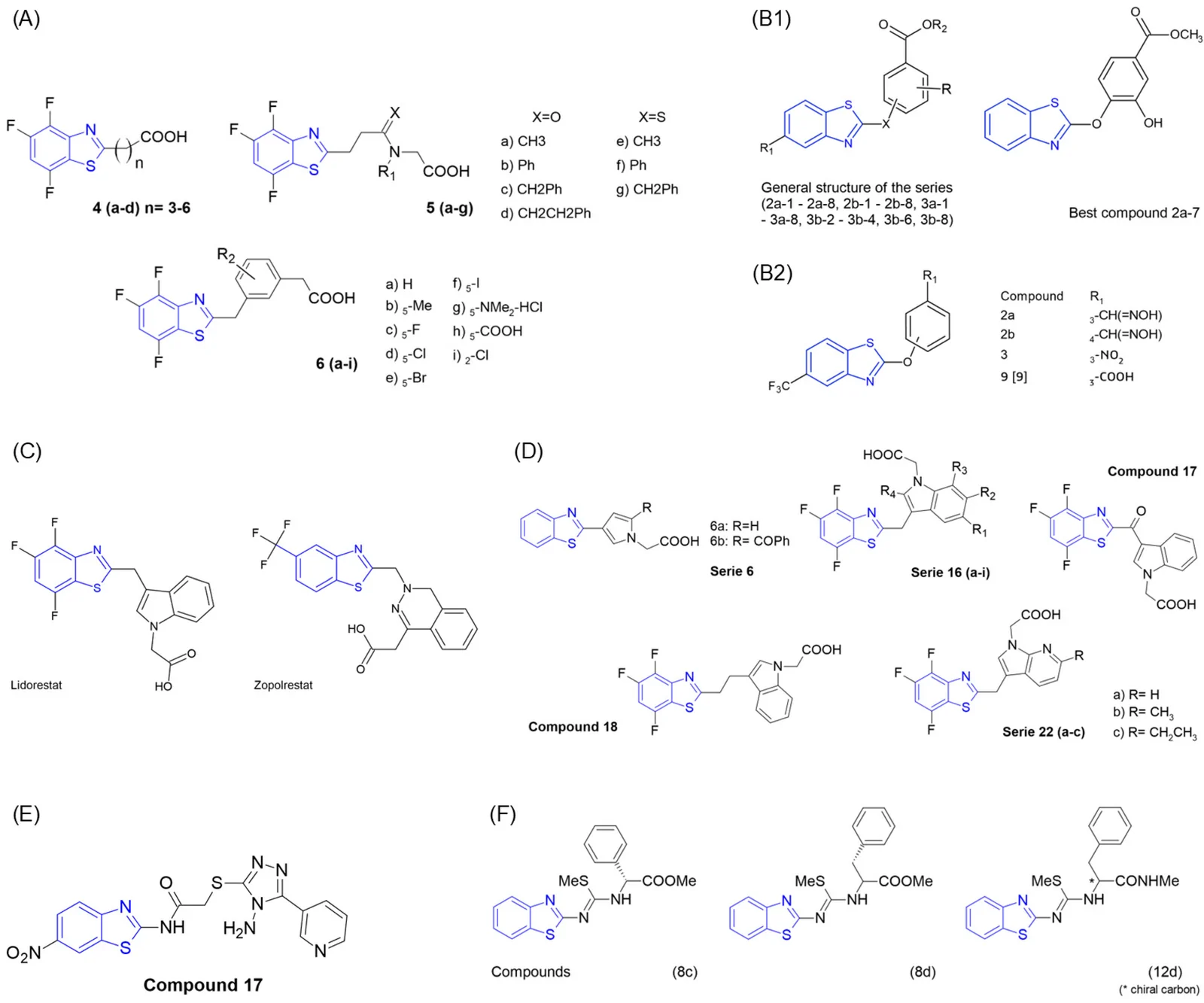
Carboxylic acid and benzothiazole were modifying the extension of the aliphatic chain in the 4(a–d). On the other hand, they modified the *N*-substituents of 5(a–g). Finally, they added a benzene 6(a–i) (**A**) [140]. Several methyl benzothiazolyloxybenzoates, S-isosters, were synthesized and tested as aldose reductase inhibitors, and the compound 2a–7 was the best (**B1**) [141]. The compounds O-benzothiazolyl oxybenzoic acids were evaluated as aldose reductase inhibitors that contained benzothiazole (**B2**) [141]. Benzothiazole-core medications (Lidorestat, zopolrestat) (**C**) [142]. 3-(Benzothiazol-2-yl) methyl]indole-N-alkanoic acids with trifluorobenzothiazole within their structure (Serie 6, (16(a–i), 18, serie 22 (a–c) and 17) (**D**) [129]. Compounds of triazoles (compound 17), which had a benzothiazole-derived substituent (**E**) [86]. Compounds 60 and 61 that contain benzothiazoles (**F**) [117].

**Figure 14:**
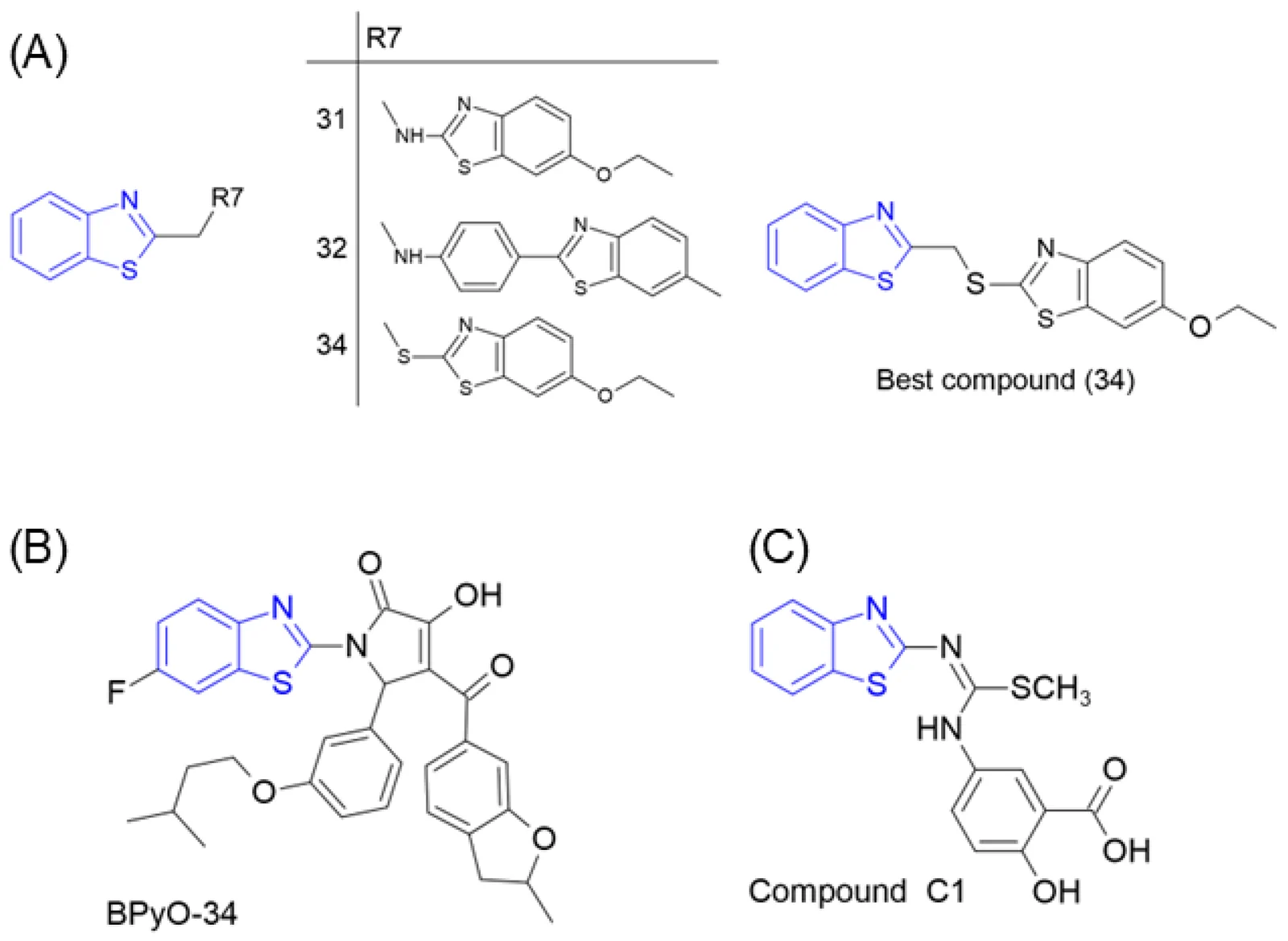
Compounds of benzothiazole derivatives targeting the AMPK activator PT-1 (**A**) [89]. 1-(6-Fluoro-benzothiazole-2-yl)-3-hydroxy-5-[3-(3-methyl-butoxy)-phenyl]-4-(2-methyl-2,3-dihydro-benzofuran-5-carbonyl)-1,5-dihydro-pyrrole-2-one (BPyO-34) (**B**) [143]. Compound C1 that contains benzothiazole in its chemical structure (**C**) [144].

**Table 1:** Representative benzothiazole-derived compounds, molecular targets, experimental evidence, and key findings.

Molecular Target	Representative Derivatives	Evidence and Experimental Model	Main Findings	Ref.
a-Glucosidase	6s, benzothiazole-triazole derivative	In vitro enzyme inhibition; in silico molecular docking	Most active compound of the series (IC_50_ = 20.7 μM). Activity was associated with a 5-Cl substituent on the benzothiazole ring and a *para*-tert-butyl group on the phenyl ring.	[12]
DPP-4	12v, 12x, and 12u	In vitro enzyme inhibition and metabolic stability; in vivo studies in rats and mice.	Compounds 12v and 12x exhibited DPP-IV inhibition in the subnanomolar range (IC_50_ = 0.64 and 0.79 nM, respectively). Compound 12u showed favorable metabolic stability and dose-dependent inhibition of plasma DPP-4 activity following oral administration.	[113]
PPAR-γ/ALR2	8c and 8d	In silico molecular evaluation; in vitro ALR2 inhibition; in vivo diabetic rat model induced by high-fat diet and streptozotocin (STZ)	The compounds inhibited ALR2 and exhibited favorable in silico characteristics regarding PPAR-γ. In vivo, they improved glycemic control and insulin resistance; compound 8d displayed the most consistent overall metabolic profile.	[117]
PPAR-α/PPAR-γ	(S)-44b	In vitro receptor assays; in vivo metabolic evaluation	The optimized stereoisomer acted as a potent full PPAR-γ agonist and a partial PPAR-α agonist, with favorable metabolic effects in vivo.	[124]
PTP1B	NM-14	In vitro PTP1B inhibition; in vivo antidiabetic evaluation	NM-14 exhibited an IC_50_ of 1.88 μM against PTP1B and antidiabetic activity comparable to that of metformin and glimepiride in the experimental model used.	[132]
Glycogen phosphorylase	2-(β-D-glucopyranosyl)-benzothiazole analogue	In vitro enzyme kinetics; X-ray crystallography	The benzothiazole analogue exhibited competitive inhibition of GPb, and its enzyme ligand binding interactions were characterized via crystallographic studies.	[134]
11β-HSD1	Compounds 1 and 2 (5-arylsulfonamido benzothiazoles)	In vitro human liver microsomes; in silico molecular docking	Both derivatives showed greater than 80% inhibition at 10 μM. Molecular docking studies supported the existence of interactions within the hydrophobic region of 11β-HSD1.	[137]
ALR2	6a, 6i, and GP-1447	Inhibition of ALR2 in vitro; STZ-induced diabetic rat model in vivo	The series demonstrated ALR2 inhibition in the nanomolar range. Compound 6i exhibited potent in vivo inhibition of sorbitol accumulation, whereas GP-1447 combined high potency with favorable oral absorption, low toxicity, and considerable selectivity.	[140]
AMPK	Compound 34	L6 myotubes in vitro; KKAy diabetic mice in vivo	Compound 34 increased glucose uptake and GLUT4 translocation through AMPK activation and reduced glucose levels in diabetic mice.	[89]
ASK1	Compounds 27 and 34	In silico/QSAR virtual screening; in vitro enzyme inhibition	Compounds 27 and 34 were the most active members of the series (IC_50_ = 2.5 and 0.52 μM, respectively). The benzothiazole core contributed to hydrophobic and π-sulfur interactions at the ASK1-binding site.	[143]
*β*3-ADR	C1	In silico molecular docking; evaluation in obese Zucker rats	C1 reduced body weight gain and modulated metabolic, inflammatory, and oxidative stress parameters through effects associated with the activation of *β*3-adrenergic receptors (*β*3-ADR), while also showing potential implications for glucose homeostasis.	[144]

## Data Availability

All data used in this review can be found in the references.

## Abbreviations

The following abbreviations are used in this manuscript:

ADA	American Diabetes Association
ADP	Adenosine diphosphate
AGE	Advanced glycation end products
ALR2	Aldose reductase
AMPK	AMP-activated protein kinase
AP-1	Protein-1 activator
ATP	Adenosine triphosphate
Br	Bromine
11β-HSD	11β-Hydroxysteroid dehydrogenase
CAT	Catalase
Cl	Chlorine
CLX	Clobenzorex
CRP	C-reactive protein
DM	Diabetes Mellitus
DNA	Oxidative alterations in deoxyribonucleic acid
EGR-1	Early-response growth factor
ER	Endoplasmic reticulum
F	Fluorine
GA	Golgi apparatus
GCK	glucokinase
GLUT	Glucose transporter
GPx	Glutathione peroxidase
GRed	Glutathione reductase
∆G	Binding energy
HO	Hydroxyl radical
h	Hour(s)
IDF	International Diabetes Federation
IL-6	Interleukin 6
IR	Insulin receptor
Ki	Inhibition constant
LD_50_	Median lethal dose
MAPK/Akt	Mitogen-activated protein kinase
mRNA	Gene expression, messenger ribonucleic acid
MTT	3-(4,5-Dimethylthiazol-2-yl)-2,5-diphenyltetrazolium bromide
NADPH	Reduced nicotinamide adenine dinucleotide phosphate
NF-κB	Nuclear factor kappa B
PI3K	Phosphatidylinositol-3-kinase
O2	Superoxide anion radical
PGZ	Pioglitazone
PKC	Protein kinase C
PPAR	Peroxisome proliferator-activated receptor
PTP-1B	Protein tyrosine phosphatase 1B
ROS	Reactive oxygen species
Ser/Thr	Phosphorylation state in serine/threonine
RSG	Rosiglitazone
SOD	Superoxide dismutase
STZ	Streptozotocin
T1DM	Type 1 diabetes mellitus
T2DM	Type 2 diabetes mellitus
TNF-alpha	Tumor necrosis factor-alpha
VEGF	Vascular endothelial growth factor
WHO	World Health Organization
IC50	Half maximal inhibitory concentration
